# Small Extracellular Vesicles in Cardioprotection, Cardiac Repair, and Regeneration: Cargo Mechanisms, Producer Cell Sources, and Translational Development

**DOI:** 10.3390/biomedicines14091989

**Published:** 2026-09-03

**Authors:** Chongyu Zhang, Prakasha Kempaiah, David J. Rademacher, W. Keith Jones

**Affiliations:** 1 Department of Molecular Pharmacology and Neuroscience, Stritch School of Medicine, Loyola University Chicago, Maywood, IL 60153, USA; czhang13@luc.edu (C.Z.); pkempaiah@luc.edu (P.K.); 2 Department of Microbiology and Immunology and Core Microscopy Facility, Stritch School of Medicine, Loyola University Chicago, Maywood, IL 60153, USA; drademacher@luc.edu

**Keywords:** cardiovascular disease, exosome, small extracellular vesicles, cardiac repair, cardioprotection, miRNA, cargo mechanisms, biomaterial-assisted delivery

## Abstract

Cardiovascular diseases remain a major cause of death and disability, and the limited regenerative capacity of the adult myocardium continues to constrain recovery after myocardial infarction, ischemia/reperfusion injury, cardiomyopathy, and heart failure. Small extracellular vesicles (sEVs), often described as exosome-enriched vesicle preparations, have emerged as promising cell-free mediators of cardioprotection and cardiac repair. This review evaluates the current experimental and translational evidence on sEVs in cardiac injury, including regulatory RNA and non-RNA cargo mechanisms, producer cell sources, preclinical injury models, engineered and biomaterial-assisted delivery strategies, and the development of clinically viable cell-free therapies. Across preclinical models, sEVs derived from mesenchymal stem cells, induced pluripotent stem cell-derived cardiac cells, cardiac progenitor cells, endothelial cells, cardiomyocytes, immune cells, and other sources have been associated with improved ventricular function, reduced infarct or scar size, enhanced angiogenesis, lower apoptosis, attenuated fibrosis, and modulation of post-injury inflammation. These effects are frequently associated with changes in, or enrichment of, microRNAs, long non-coding RNAs, and circular RNAs that regulate survival signaling, immune polarization, extracellular matrix remodeling, and endothelial recovery. Emerging evidence further suggests that proteins, lipids, and organelle-associated cargo, including mitochondrial components, also contribute to stress adaptation, vesicle biogenesis, mitochondrial homeostasis, and metabolic repair. However, the field remains limited by inconsistent nomenclature, variable isolation and characterization methods, heterogeneous producer cell sources, uncertain potency assays, incomplete biodistribution data, and unresolved regulatory classification. Overall, sEV-based therapy represents a compelling but still developing approach to cardiac repair. Within this framework, functional recovery is treated as an outcome of cardioprotection, cardiac repair, or, where supported, myocardial regeneration; regeneration is reserved for evidence of newly formed and functionally integrated myocardium. Future progress will depend on better product definition, mechanism-linked potency testing, scalable manufacturing, and delivery strategies matched to specific cardiac indications.

## 1. Introduction

### 1.1. Myocardial Infarction and Cardiac Failure

Cardiovascular diseases (CVDs) continue to be the leading cause of mortality worldwide, with the highest burden in low- and middle-income countries [1]. Although age-standardized CVD mortality has declined in some settings, CVD still causes approximately 19.2 to 20.5 million deaths annually, leading to an increased burden of disability and reduced quality of life [1,2]. Acute and chronic cardiac injuries include myocardial infarction (MI), ischemia/reperfusion (I/R) injury, cardiomyopathies, and heart failure, characterized by extensive loss of cardiomyocytes, microvascular dysfunction, ongoing inflammation, and pathological remodeling [2]. Endogenous repair mechanisms are frequently insufficient to restore myocardial structure and function due to the limited regenerative capacity of adult cardiomyocytes, underscoring the urgent need for therapeutic strategies that promote cardioprotection and cardiac repair and, ultimately, achieve true myocardial regeneration [3]. Understanding the molecular underpinnings of cardiac remodeling is a challenge in cardiovascular medicine because, in contrast to other tissues, the heart has very little capacity for self-regeneration. Billions of cardiomyocytes are lost after an acute event such as an MI, and non-contractile fibrotic scar tissue takes their place [4]. This pathological remodeling results in heart failure, a condition for which the available pharmaceutical and surgical treatments are only palliative, slowing the disease’s progression without repairing the damaged cells.

### 1.2. The Evolution from Cell-Based to Cell-Free Therapy

Stem cell transplantation (e.g., cardiac progenitor cells, mesenchymal stem cells) has been a major focus of regenerative cardiology for the last 20 years. Poor cell retention, low engraftment rates, potential immunogenicity, tumorigenesis or arrhythmias, and difficulties with cell sourcing and manufacturing have hindered clinical translation, despite early preclinical studies showing functional improvements [5]. According to recent data, the positive outcomes seen in stem cell-based trials may have resulted from the paracrine effects of the transplanted cells—the release of bioactive molecules that alter the local microenvironment—rather than from the cells differentiating into new heart muscle [6]. Consequently, the focus has shifted to cell-free therapeutic approaches, especially those that use extracellular vesicles (EVs), frequently enriched in vesicles known as exosomes, as important mediators of the paracrine effects previously linked to transplanted cells [7,8].

### 1.3. Small Extracellular Vesicles

Small extracellular vesicles (sEVs), often described in earlier studies as exosomes or exosome-enriched vesicle preparations, have shown promise as biological vectors that mimic many of the cardioprotective and reparative effects of parent cells without the dangers of live cell transplantation [9]. They are membrane-bound, nanoscale vesicles that are released by almost all cell types and are becoming more widely acknowledged as essential mediators or regulators of intercellular communication. sEVs can carry selectively enriched molecular cargo as paracrine signaling molecules, including proteins, lipids, metabolites, functional mitochondrial components, and regulatory RNAs like microRNAs (miRNAs), circular RNAs (circRNAs), and long non-coding RNAs (lncRNAs) [10]. In the cardiovascular system, sEVs derived from various cells (e.g., stem cells, endothelial cells (ECs), cardiomyocytes, immune cells, and fibroblasts) have been demonstrated to impact important biological processes central to cardioprotection and repair, including apoptosis inhibition, angiogenesis, immune modulation, oxidative stress regulation, fibrosis attenuation, and metabolic adaptation [11]. In the context of cardiac therapy, stem cell-derived sEVs provide a “cell-free” therapeutic approach while emulating the therapeutic effects of the parent cells [12]. This offers distinct advantages including higher stability, lower immunogenicity, lack of tumorigenic risk, and the potential for off-the-shelf pharmaceutical manufacturing.

### 1.4. Role of Regulatory RNAs

Among these candidate cargoes, regulatory RNAs have received particular attention [13]. These non-coding RNAs (ncRNAs) are master regulators of gene expression. They can simultaneously target several signaling pathways to change the course of heart disease when administered via sEVs to damaged cardiac tissue [14]. Regulatory RNAs have attracted particular interest due to their potent and pleiotropic effects on gene expression networks governing cardiac homeostasis and injury responses [15]. While circRNAs and lncRNAs function as miRNA sponges, transcriptional modulators, and scaffolding molecules that affect chromatin dynamics and cellular phenotype, miRNAs can fine-tune post-transcriptional gene regulation involved in cardiomyocyte survival, endothelial function, and inflammatory signaling [16]. Therefore, increasing evidence suggests that the therapeutic efficacy of sEVs in cardioprotection and cardiac repair is largely linked to RNA-associated pathways, positioning regulatory RNAs as both functional effectors and potential biomarkers of cardiac repair processes.

### 1.5. Three Therapeutic Pillars of sEV-Based Cardiac Therapy

According to current research, sEV therapy may support cardiac recovery through three biological pillars: (1) cardioprotection, which limits acute ischemic and I/R injury by inhibiting apoptotic and inflammatory cell death pathways in stressed cardiomyocytes and supporting early survival signaling; (2) cardiac repair, which modifies the post-injury tissue environment by shifting macrophage states toward inflammatory resolution, suppressing pathological fibrosis, promoting angiogenesis, and supporting vascular recovery; and (3) myocardial regeneration, which should be reserved for evidence of meaningful restoration of lost myocardium, including sustained cardiomyocyte replacement, durable cell cycle completion, or other evidence beyond improved function or reduced scar alone. Recovery is best understood as the functional and structural outcome of these processes rather than as a separate biological pillar.

The remainder of this review is organized around this framework. Section 4 examines the biological mechanisms underlying cardioprotection and cardiac repair and evaluates the evidentiary threshold for myocardial regeneration. Section 5 compares EV products according to producer cell source. Section 6 and Section 7 evaluate RNA and non-RNA cargo, respectively, and Section 8 examines therapeutic outcomes across models.

In a variety of experimental models of cardiac injury, preclinical research has shown that sEV administration can limit adverse remodeling, increase neovascularization, decrease infarct size, and improve cardiac function [17]. Furthermore, advances in bioengineering have enabled the modification of sEV cargo, surface ligands, and delivery strategies to enhance cardiac targeting and therapeutic potency. These features, combined with their relative stability, low immunogenicity, and scalability, make sEVs highly attractive candidates for next-generation, cell-free cardiovascular therapies [18]. These observations support continued development of cell-free cardiovascular therapies.

Despite this growing body of evidence, significant challenges remain in translating sEV-based therapies into clinical practice. Considerable heterogeneity exists across studies with respect to the source of vesicles, isolation and characterization methods, RNA profiling techniques, and dosing regimens [19]. Furthermore, the relative contributions of different regulatory RNA species to cardioprotection, cardiac repair, and functional recovery-and whether any contribute to myocardial regeneration-remain incompletely resolved. Therefore, a comprehensive synthesis of the existing literature is essential to clarify the mechanistic roles of sEV-associated regulatory RNAs and to evaluate the therapeutic potential of sEV-based, cell-free strategies for CVDs. With a focus on RNA-mediated mechanisms and their implications for the development of clinically feasible EV-based therapies, this review critically evaluates the available experimental and translational data on sEVs in cardioprotection, cardiac repair, functional recovery as a measured outcome, and evidence claimed to support myocardial regeneration.

Recent reviews have addressed specific components of this field, including MSC-derived sEV miRNAs in heart repair and regeneration [20], the biological and therapeutic roles of EVs in acute myocardial infarction (AMI) [21], biomaterial-assisted EV delivery in preclinical heart failure models [22], and mitochondria-enriched EVs for restoring cardiac bioenergetics [23]. Building on these focused syntheses, the present review integrates EV source and biogenesis, regulatory RNA and organelle cargo, injury response mechanisms, model-specific outcomes, and translational barriers across cardioprotection, cardiac repair, recovery, and regeneration.

## 2. Methods

A predetermined framework outlining specific study characteristics was used to screen and choose studies for inclusion in this narrative review. The inclusion criteria included: (1) peer-reviewed original research articles or comprehensive literature reviews; (2) cardiac-relevant applications using sEVs or exosomes; and (3) models spanning translational systems, in vivo animal models, and in vitro cellular assays. The following were the criteria for exclusion: (1) research assessing applications that are solely non-cardiac; (2) investigations that solely isolated or focused exclusively on large EVs; and (3) literature without peer review, such as book chapters, editorials, conference abstracts, and commentary. Three electronic databases—PubMed, Scopus, and Web of Science—were used in a thorough, methodical literature search to find works published between January 2005 and January 2026. In order to find pertinent proprietary and translational technologies within the same time frame as the primary database searches, a special patent search was conducted on 8 February 2026, using Google Patents, Espacenet, and WIPO PATENTSCOPE. The search was designed to capture patent records in which EVs or exosomes were used as therapeutic agents, active components, delivery systems, or engineered biologic products for cardiac injury, ischemic heart disease, myocardial infarction, I/R injury, heart failure, arrhythmia, myocarditis, cardiomyopathy, cardioprotection, cardiac repair, or cardiac regeneration. The search terms combined three main concept groups: EV-related terms, cardiac disease or injury terms, and therapeutic effect terms. The core search strategy included the following terms, adapted as needed for each patent database: (“exosome” OR “extracellular vesicle” OR “EV” OR “microvesicle” OR “sEV” OR “ectosome”) AND (“cardiac” OR “heart” OR “myocard*” OR “myocardial infarction” OR “ischem*” OR “ischemia reperfusion” OR “heart failure” OR “HFpEF” OR “arrhythm*” OR “myocarditis” OR “cardiomyopath*”) AND (“treat*” OR “therap*” OR “repair” OR “regenerat*” OR “cardioprotect*”). Additional searches incorporated source cell and product development terms, including mesenchymal, MSC, cardiosphere-derived, cardiac stromal, cardiac progenitor, cardiomyocyte, and induced pluripotent stem cell (iPSC). A separate delivery-focused search incorporated terms related to stents, patches, hydrogels, scaffolds, catheters, targeting peptides, E-selectin, and P-selectin. Patent records were deduplicated at the patent family level when possible. Patent families were retained when EVs or exosomes were described as a required therapeutic agent, active component, or delivery platform for an explicit cardiac indication. Records were excluded when EVs were used only for diagnostic purposes, mentioned only incidentally, or listed within broad non-specific regenerative claims without a clear cardiac therapeutic embodiment. To guarantee literature saturation, the reference lists of all retrieved primary articles and pertinent reviews were manually cross-checked. The precise search string structure that was applied to all databases was “exosomes” OR “small extracellular vesicles” AND “cardiac” AND (“regeneration” OR “repair” OR “protection”). EndNote 20.1 software was used to compile the results of the literature search and electronically eliminate duplicate entries. After this, full-text versions of all potentially pertinent studies were obtained.

## 3. Extracellular Vesicle Nomenclature, Biogenesis, and Characterization

### 3.1. Nomenclature and Biogenesis

EVs are membrane-bound particles that are released by the majority of cell types and are becoming more widely acknowledged as crucial intercellular communication mediators in both health and disease conditions [24,25,26]. EVs have garnered special attention in the cardiovascular setting as their associated cargo and surface signals have been linked to cardiomyocyte survival, vascular repair, inflammation, fibrosis, and post-injury remodeling [24,25,26,27]. The main vesicle population discussed herein is the sEV fraction, which is operationally defined as being smaller than 200 nm and frequently enriched for vesicles known as exosomes [24]. The definition of these vesicles, however, varies in the cardiac therapeutic literature. Without providing concrete proof of endosomal origin, many studies characterize exosomes mainly based on size, enrichment workflow, and marker expression. Because of this, and in compliance with the Minimal Information for Studies of Extracellular Vesicles 2023 (MISEV2023) guidelines, operationally defined small vesicle preparations should be referred to as sEVs, while vesicles more closely associated with the endosomal pathway should be referred to as exosomes [24]. In this review, the term EV or sEV is used unless endosomal origin is proven.

Biogenesis is the main factor that distinguishes exosomes from microvesicles. Exosomes originate from the endosomal system through formation of intraluminal vesicles within multivesicular bodies (MVBs), followed by fusion of those bodies with the plasma membrane and extracellular release [25,26]. By contrast, microvesicles are generated by direct outward budding and fission from the plasma membrane [25]. These biogenetic variations could, in theory, influence biological behavior, membrane composition, and cargo loading. However, in reality, the majority of post-isolation cardiac EV preparations cannot be definitively attributed to a single intracellular origin, especially when they come from complex biofluids that contain mixed vesicle populations or conditioned media. Therefore, the use of the term sEV reflects the practical limitations of the field and provides a more rigorous framework for interpretation [24,26].

### 3.2. Characterization Standards and Marker Panels

Particle size is a similar problem. Vesicles of interest are described as being smaller than 200 nm in a large portion of the cardiac literature, which is consistent with exosome-enriched or sEV fractions [24,26]. However, vesicle identity cannot be determined solely by size. Particles in the anticipated size range are not always endosome-derived, and size does not rule out contamination by lipoproteins, protein aggregates, or other non-EV material. Therefore, compared to studies that use multiple complementary approaches, those that solely rely on particle size without biochemical and morphologic characterization offer weaker evidence for vesicle identity [24,28]. This is especially important in cardiovascular research, where the composition and purity of the isolated vesicle fraction can have a significant impact on how therapeutic effects are interpreted.

Therefore, marker-based characterization is crucial, but it has drawbacks. Tetraspanins, like CD63 and CD9, as well as proteins linked to endosomal sorting and vesicle formation, like tumor susceptibility gene 101 protein (TSG101) and ALG-2 interacting protein X (ALIX), are some of the most widely used EV markers. Although these markers are essential for the current characterization of EVs, none of them alone can adequately establish the identity of exosomes. While CD9 is frequently employed as an EV membrane marker, CD63 is frequently linked to late endosomal/MVB-related EV populations; neither is exosome-specific [24,25,26]. Similarly, TSG101 and ALIX are linked to vesicle biogenesis pathways and are helpful markers of intracellular sorting machinery associated with vesicles [24,26]. None of these markers, alone or together, can indisputably define vesicle origin. MISEV2023 recommends that positive markers should ideally be accompanied by evidence excluding major contaminants [24]. This point is particularly crucial because the degree to which vesicle quality is documented varies greatly between studies, and the strength of subsequent mechanistic claims is partially dependent on that underlying rigor.

### 3.3. Isolation Strategies and Their Interpretive Consequences

Another significant layer of variability is added by isolation strategy. Due to its familiarity, accessibility, and compatibility with large sample volumes, differential ultracentrifugation has historically been one of the most popular techniques and is still frequently used in the cardiac EV literature [29]. However, ultracentrifugation also has important limitations. Repeated high-speed centrifugation may cause particle aggregation or structural damage, and it can co-isolate protein aggregates and other non-vesicular material [24,28]. These concerns were specifically addressed in the context of cardiac regeneration by Angelini et al. [28], who also emphasized how isolation artifacts could make it more difficult to interpret reports of vesicle-mediated repair. These issues are more than just small technical details. Isolation artifacts can have a direct impact on biological interpretation in a therapeutic literature where downstream claims frequently focus on cargo composition and repair activity [28].

The cardiac EV literature also includes commercial precipitation-based workflows. For instance, in an MI study, hypoxia-conditioned human mesenchymal stem cell (MSC)-derived sEVs were isolated using a Total Exosome Isolation reagent kit [30]. These reagent-based methods are appealing due to their ease of use and the lack of need for specialized ultracentrifugation equipment, but they also raise additional concerns regarding the co-isolation of soluble proteins and other non-vesicular material. Precipitation-based preparations can, therefore, be helpful in accessibility-driven or discovery-stage research, but before firm cargo-specific or mechanism-specific conclusions are made, they need particularly careful downstream characterization [24,28,30]. Because of this, the isolation protocol employed must be taken into consideration when interpreting the results of both older and more recent studies.

Filtration-based and hybrid purification processes have become more widely accepted in recent years. These include ultrafiltration (UF), tangential flow filtration (TFF), and filtration combinations with size exclusion chromatography (SEC) in cardiac-relevant systems [31,32,33]. Andrade et al. [31] reported using TFF to produce EVs derived from human iPSCs with better EV recovery than TFF followed by ultracentrifugation. This demonstrates how both vesicle yield and downstream functional readouts can be impacted by preparation mode. Filtration is frequently integrated into multistep workflows rather than being considered a full replacement for orthogonal cleanup, as demonstrated by other studies that used TFF plus SEC or UF plus SEC [32,33].

As an alternative, SEC and immunoaffinity isolation can mitigate some of these limitations, but they also come with drawbacks [24]. When downstream mechanistic or functional assays demand greater purity, SEC is appealing because it can more effectively separate vesicles from soluble proteins and some non-vesicular contaminants while maintaining vesicle integrity [24,34]. When a more selective vesicle subpopulation is required, immunoaffinity techniques can be helpful because they enrich vesicles based on surface proteins like tetraspanins [24]. Nevertheless, by favoring marker-positive subpopulations, these techniques also introduce bias and might not accurately reflect the total pool of secreted vesicles. Therefore, there is not a single isolation technique that is always better. Instead, a study’s interpretive strength is determined by how well its isolation strategy aligns with its experimental goal and whether orthogonal characterization supports it [24,28,31,33,34].

These concepts are demonstrated by a number of cardiac-focused studies. In addition to describing the isolation and characterization of EVs secreted from cardiovascular progenitor cells derived from human pluripotent stem cells, Wu et al. [35] offered a helpful illustration of how vesicle collection, particle sizing, and marker-based confirmation can be combined in a cardiac cell system that is therapeutically relevant. Zwi-Dantsis et al. [34] highlighted the significance of purification quality when interpreting cell type specificity with highly purified EVs from human cardiomyocytes and their demonstration of preferential uptake by human ECs. Kang et al. [36] examined the EVs of human cardiac mesenchymal stromal cells and bone marrow-derived mesenchymal stem cells (MSCs), further demonstrating that vesicle characterization is most instructive when interpreted in conjunction with the producer cell’s phenotype. By connecting MSC-derived sEV characterization with translational imaging outcomes, Charles et al. [37] expanded this reasoning to a porcine MI model, highlighting the fact that characterization is not only descriptive but fundamental to therapeutic interpretation.

In cardiac disease, biogenesis itself might also be dynamically relevant. Using transgenic mice with cardiac-specific heat shock protein 20 (Hsp20) overexpression, Wang et al. [38] demonstrated that Hsp20 interacted with TSG101, increased cardiomyocyte sEV production, and improved cardiac function and angiogenesis in diabetic mice. This study is important as it suggests that EV biogenesis is not a static background process but can be regulated by the pathophysiological state of the producer cells [38]. Thus, biogenesis, nomenclature, and characterization are closely linked, and each affect how the cardiac EV literature should be interpreted.

Taken together, the studies included in this review support several practical conclusions. First, unless endosomal origin has been proven, the most rigorous term for the vesicle populations discussed herein is sEV [24]. Second, rather than relying solely on size, vesicle identity is best supported by morphology, size distribution, positive marker enrichment, and contaminant-aware characterization [24,25,26,28]. Third, isolation strategy has a significant impact on purity, yield, and interpretability; therefore, when the upstream and downstream workflows differ, nominally similar EV preparations may not be directly comparable across studies [24,28,29,34]. These issues are not peripheral. They determine how confidently one can interpret reported cargo composition, assign biological activity to vesicles rather than co-isolated material, and evaluate claims of mechanism or therapeutic efficacy in the injured heart. Therefore, before thinking about the mechanistic pathways covered in Section 4 below, careful attention to nomenclature and characterization is crucial.

## 4. Therapeutic Mechanisms in Cardiac Injury

**Figure 1:**
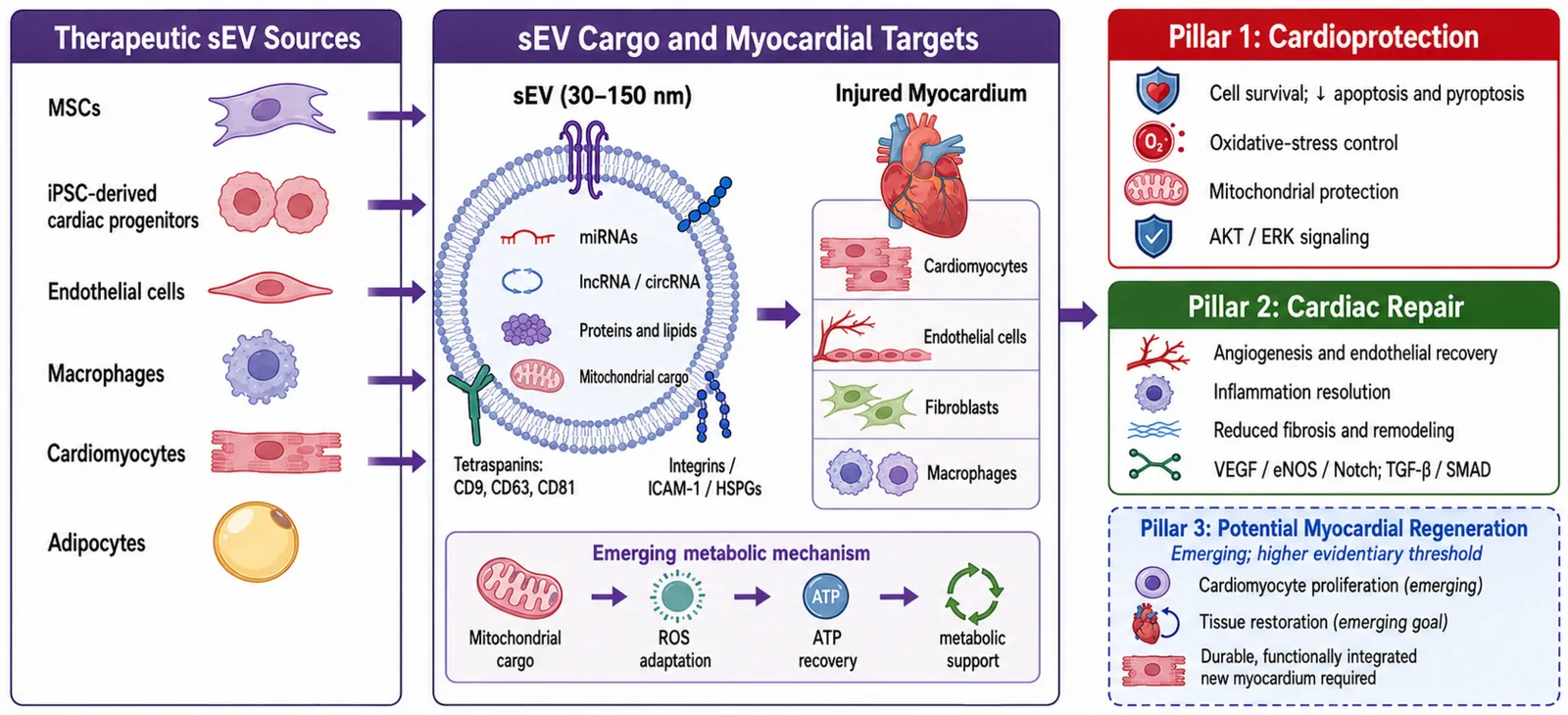
A three pillar framework for sEV-mediated cardiac therapy. Therapeutic sEV sources include MSCs, cardiac progenitor/iPSC-derived progenitor cells, ECs, macrophages, cardiomyocytes, and adipocytes. The sEV contains tetraspanins CD9, CD63, CD81, integrins, ICAM-1, adhesion molecules, heparan sulfate proteoglycans, and lipid raft domains. sEV cargo includes miRNAs, lncRNAs, circRNAs, proteins, lipids, and mitochondria/metabolic cargo. The cargo is proposed to act on cardiomyocytes, ECs, fibroblasts, and macrophages. Therapeutic outcomes and mechanisms include cardioprotection, cardiac repair, and myocardial regeneration. Within the cardioprotection pillar, the proposed outcomes include lower apoptosis, pyroptosis, oxidative stress, and mitochondrial injury. Within the cardiac repair pillar, the proposed outcomes include endothelial recovery and angiogenesis, inflammatory resolution, fibrosis restraint, and improved remodeling. Regeneration is shown as a more stringent and emerging goal requiring evidence beyond improved function or reduced scar burden. Candidate pathways include AKT/ERK, VEGF/eNOS/Notch, TGF-β/SMAD, M1-to-M2 macrophage shift, reduced apoptosis, reduced pyroptosis, reduced fibrosis, and reduced inflammatory signaling. Abbreviations: sEV, small extracellular vesicle; MSC, mesenchymal stromal cell; iPSC, induced pluripotent stem cell; miRNA, microRNA; lncRNA, long non-coding RNA; circRNA, circular RNA; ICAM-1, intercellular adhesion molecule 1; HSPG, heparan sulfate proteoglycan; ROS, reactive oxygen species; ATP, adenosine triphosphate; AKT, protein kinase B; ERK, extracellular signal-regulated kinase; VEGF, vascular endothelial growth factor; eNOS, endothelial nitric oxide synthase; TFG-β, transforming growth factor-beta; SMAD, suppressor of mothers against decapentaplegic. This figure was created with BioRender.com. http://app.biorender.com/ (accessed on 28 June 2026).

As shown in Figure 1, therapeutic EVs, and especially sEVs, seem to enhance cardiac repair through several recurrent and partially overlapping mechanisms. Within the three pillar framework described above (Section 1.5), four recurring biological mechanistic themes—cell death limitation, angiogenesis, fibrosis/remodeling control, and immune modulation—consistently emerge across the preclinical literature. Cardioprotection primarily concerns the preservation of viable myocardium during or acutely after injury. Cardiac repair concerns restoration of the post-injury tissue environment through vascular recovery, inflammatory resolution, fibrosis restraint, and control of adverse remodeling. Regeneration is the most stringent therapeutic goal and requires evidence beyond improved ventricular function or reduced scar burden alone.

The mechanisms described herein are not mutually exclusive. Anti-apoptotic and anti-pyroptotic effects can preserve viable myocardium available for later repair, while immune resolution can influence angiogenesis, fibrosis, and longer-term remodeling. The following subsections distinguish the three therapeutic pillars for clarity, while recognizing that individual biological pathways may contribute to more than one outcome.

### 4.1. Cardioprotection: Limiting Acute Cardiomyocyte Injury

Protection against cardiomyocyte death is one of the clearest and most frequently reported effects (Figure 1). EVs derived from various producer cell types decrease apoptosis and maintain myocardial viability in models of MI, I/R, and other cardiac injuries [39,40,41,42,43,44,45,46]. The strongest evidence is found when vesicle treatment is linked to reduced cell death markers, activation of identifiable survival pathways, and improved function.

The work of Luther et al. [41] remains a representative cargo-linked example of this pillar. MSC-derived sEVs mediated cardioprotection, in part, through miR-21a-5p, which was linked to the repression of multiple pro-apoptotic targets, including programmed cell death protein 4 (PDCD4), phosphatase and tensin homologue (PTEN), pellino E3 ubiquitin protein ligase 1 (PELI1), and Fas ligand (FasL) [41].

Although many of these studies are better understood as pathway-associated rather than equally direct cargo-causal evidence, they nonetheless support the wider significance of survival signaling. Through miR-221-mediated targeting of PTEN and subsequent phosphoinositide 3-kinase/protein kinase B (PI3K/AKT) signaling, GATA binding protein 4 (GATA4)-enhanced sEVs from cardiac colony-forming unit fibroblasts exerted anti-apoptotic effects [42]. Human umbilical vein endothelial cell (HUVEC)-derived sEVs and ischemic preconditioning-induced serum EVs were also associated with PI3K/AKT activation and improved cardiac injury outcomes [39,40]. Extracellular signal-regulated kinase (ERK)-dependent protection has also been implicated, including in dystrophin-deficient cardiomyocytes and hydrogen peroxide-stressed H9C2 cells [43,44]. Together, these findings imply that coordinated suppression of multiple injury pathways, as opposed to a single signaling node, is frequently how EV-mediated cardioprotection is accomplished [39,40,41,42,43,44].

Beyond traditional apoptosis, cardiomyocyte preservation also involves inflammatory cell death programs like pyroptosis. While M2 macrophage-derived sEVs attenuated reperfusion injury via the reactive oxygen species (ROS)/NOD-like receptor protein 3 (NLRP3) axis [47], MSC-derived sEVs decreased myocardial I/R injury by blocking pyroptosis [48]. Toll-like receptor 4 (TLR4)/NLRP3-mediated pyroptotic injury in doxorubicin cardiotoxicity was also inhibited by sEVs derived from embryonic stem cells (ESC) [49]. More recent work extends this idea to immunotherapy-associated cardiac injury, where human bone marrow MSC-derived sEVs alleviated programmed cell death protein 1 (PD1)/programmed cell death-ligand 1 (PD-L1) inhibitor-associated myocardial injury through effects on macrophage polarization and pyroptosis [50]. These findings support the idea that EVs can modulate both apoptotic and inflammatory forms of cardiomyocyte death and extend the mechanistic scope of EV therapy beyond traditional ischemic injury [47,48,49,50].

### 4.2. Cardiac Repair: Restoring the Post-Injury Myocardial Environment

#### 4.2.1. Pro-Angiogenesis and Endothelial Recovery

Promoting angiogenesis and endothelial repair is a major component of cardiac repair (Figure 1). This is one of the oldest and most consistent themes in the cardiac EV literature. Early research revealed that EVs from human bone marrow MSCs stimulated angiogenesis following MI [51]. Subsequent research revealed that sEVs from MSCs and cardiomyocyte progenitor cells could stimulate angiogenesis through extracellular matrix metalloproteinase inducer (EMMPRIN)-dependent signaling [52]. Cardiac MSC-derived sEVs have also been used directly for angiogenesis-focused therapy [53].

Additional studies support this using different cells or conditioning. Ma et al. [54] demonstrated pro-angiogenic activity in AKT-modified umbilical cord MSC sEVs via platelet-derived growth factor D (PDGF-D). Others showed that ESC-derived or cardiovascular progenitor cell-derived sEVs reduced cardiomyocyte death while promoting vascular repair and functional recovery [55,56]. Likewise, hypoxia-inducible factor 1-α (HIF-1α)-overexpressing MSC-derived sEVs enhanced cardioprotection in MI by increasing angiogenesis [57]. Notch signaling is also important in this context. The activation state of the producer cell can influence the reparative profile of the vesicles it releases, as demonstrated by the promotion of myocyte proliferation and neovasculogenesis by EVs from Notch-activated cardiac MSCs [58]. Other research supports this pro-angiogenic theme, such as cardiomyocyte-derived sEVs that activate endothelial nitric oxide synthase (eNOS) in cardiac microvascular ECs to prevent I/R injury [59] and remote ischemic conditioning-derived plasma sEVs associated with Hsp70 and enhanced angiogenesis [60]. Taken together, these studies support the view that selected therapeutic EV preparations can enhance endothelial recovery and neovascularization in injured myocardium.

Importantly, however, angiogenic effects are not inherent to EVs as a class, but rather heavily rely on producer cell identity and activation state. After MI, M2 macrophage-derived sEVs enhanced cardiac function and angiogenesis, partly by targeting thrombospondin 1 (THBS1) with miR-132-3p [61]. In contrast, M1-like macrophage-derived sEVs had the opposite effect, suppressing angiogenesis and worsening dysfunction through miR-155-dependent repression of pathways including sirtuin 1/5′-AMP-activated protein kinase catalytic subunit α-2/eNOS (SIRT1/AMPKα2/eNOS) and Ras-related C3 botulinum toxin substrate 1/p21-activated kinase 2 (RAC1/PAK2) [62]. Likewise, cardiac EVs released under inflammatory conditions support angiogenesis differently than EVs released under non-inflammatory conditions [63]. These results suggest that angiogenic benefit is a characteristic of particular vesicle preparations influenced by producer cell identity and producer cell state, and the effects of these upon cargo, rather than an inherent characteristic of EVs as a class [51,52,53,54,55,56,57,58,59,60,61,62,63].

#### 4.2.2. Anti-Fibrosis and Remodeling Control

Maladaptive remodeling and attenuation of fibrosis are additional components of cardiac repair (Figure 1). Crucially, even in cases where overall cardiac function improves, anti-fibrotic benefit should not be presumed for all vesicle preparations. This caution is warranted because fibroblast responses are highly context-dependent and may vary with source, timing, and disease setting [64].

In this regard, however, several studies reported that EV therapy improved broader structural remodeling following injury, decreased collagen deposition, or limited myofibroblast activation [65,66,67,68,69]. Human amniotic fluid MSC-derived sEVs alleviated cardiac fibrosis while enhancing angiogenesis [65], and human umbilical cord MSC-derived sEVs improved myocardial repair in association with the upregulation of SMAD family member 7 (SMAD7), a negative regulator of canonical TGF-β/SMAD profibrotic signaling [45]. Accordingly, rosmarinic acid-treated sEVs were reported to alleviate fibrosis through modulation of TGF-β1/SMAD family member 3 (SMAD3) signaling [67], while bone marrow MSC-derived sEVs reduced cardiac hypertrophy and fibrosis in pressure-overload remodeling [68]. The notion that upstream conditioning can enhance the anti-remodeling profile of therapeutic vesicles is further supported by oncostatin M-enriched sEVs and MI serum preconditioned bone marrow MSC-derived sEVs [69]. By contrast, serum EV-enriched preparations from transverse aortic constriction mice promoted myofibroblast and hypertrophic responses, whereas Tipifarnib treatment reduced circulating particle abundance and was associated with less ventricular dysfunction, hypertrophy, and fibrosis [70].

These context-dependent findings also motivate targeted engineering of remodeling pathways. A liposomal system carrying an inhibitor of EV/sEV-associated miR-185-5p was used to alleviate apoptosis and cuproptosis in dilated cardiomyopathy [71], and fibroblast activation protein aptamer-functionalized EVs enabled targeted delivery of TGF-β1 small interfering RNA (siRNA) to injured cardiac tissue, directly linking EV engineering to anti-remodeling therapy [72]. According to these studies, fibrosis control may become a translational endpoint for engineered EV systems that is more tractable. However, compared to the literature on angiogenesis and apoptosis, the literature on fibrosis is more context-dependent. According to reviews of fibroblast biology, depending on the source, context, and timing, EVs can either inhibit or stimulate fibroblast activation [64]. Therefore, rather than having a universal effect, anti-fibrotic benefit should be described as a recurrent but conditional feature of specific therapeutic preparations.

#### 4.2.3. Anti-Inflammation and Immune Modulation

Immune modulation is a major repair mechanism and serves as a link between the other repair processes in many respects (Figure 1). Therapeutic benefit depends not only on the cargo delivered by EVs, but also on whether vesicle treatment shifts the post-infarct immune environment toward resolution rather than sustained inflammatory recruitment. EV-driven modifications in immune cell phenotype may have wide-ranging downstream effects because inflammation affects vascular remodeling, scar formation, and long-term ventricular performance [33,73,74,75,76,77,78,79,80,81].

Multiple studies show that therapeutic EVs influence macrophage phenotype and inflammatory signaling after cardiac injury. While M2 macrophage-derived sEVs regulated C-C chemokine receptor type 2 positive (CCR2+) pro-inflammatory macrophage subpopulations and favored post-acute MI repair [73], cardiosphere-derived cell EVs reprogrammed inflammatory macrophages toward an arginase 1 (ARG1)-dependent pro-angiogenic phenotype [74]. Lipopolysaccharide preconditioned bone marrow MSC-derived sEVs produced stronger M2 macrophage polarization and greater attenuation of post-MI inflammation and cardiomyocyte apoptosis than sEVs from unconditioned bone marrow MSCs [77], and adipose MSC-derived sEVs promoted macrophage M2 polarization through sphingosine-1-phosphate/sphingosine kinase 1/sphingosine-1-phosphate receptor 1 (S1P/SPHK1/S1PR1) signaling [78]. Dental pulp MSC-derived EVs also modulated macrophage phenotype during both acute and chronic post-MI inflammation [33], and M2 macrophage sEVs have more recently been shown to reverse post-MI heart failure by suppressing type I interferon signaling in myeloid cells [79].

Platelet membrane-engineered EVs that hitchhike peripheral monocytes to improve targeting [80] and engineered EVs created for multi-targeted immunomodulatory therapy in viral myocarditis [81] are two examples of how this immunomodulatory logic is currently being extended into engineered systems. Other studies similarly identify immune regulation as a core mechanism by which EVs influence tissue remodeling and angiogenesis after MI [75,76]. These examples show that immune targeting is becoming part of product design. However, since inflammatory macrophage-derived vesicles can worsen injury and impede vascular repair, immune modulation is not always advantageous [62]. Thus, the immune context created by treatment promotes reparative resolution rather than chronic inflammatory amplification, which determines the therapeutic value of EVs.

### 4.3. Regeneration: An Emerging and More Stringently Defined Therapeutic Goal

Regeneration should be distinguished from the cardioprotection and repair outcomes discussed above. Reduced apoptosis, improved ejection fraction (EF) or fractional shortening, smaller scar burden, enhanced angiogenesis, and less fibrosis are important therapeutic endpoints, but they do not independently demonstrate restoration of lost myocardium. In this review, regeneration is, therefore, reserved for evidence that an intervention contributes to meaningful myocardial replacement or durable restoration of cardiomyocyte mass, including well-supported cardiomyocyte cell cycle re-entry and completion, tissue integration, or comparable evidence of newly formed functional myocardium. Accordingly, regeneration should remain an evidence-level claim rather than a presumed consequence of stem cell-derived EV therapy. It is treated herein primarily as an evidentiary benchmark for interpreting reported EV outcomes, rather than as a comparably established mechanism category.

### 4.4. RNA-Mediated Regulation as a Cross-Cutting Cargo Mechanism

Regulatory RNA cargo may contribute across the cardioprotection and cardiac repair pillars by influencing recipient cell survival, endothelial recovery, inflammation, fibrosis, and remodeling. However, the identification of a candidate RNA in an EV preparation or an increased RNA signal in recipient cells does not, by itself, establish direct functional cytosolic transfer. Section 6 examines RNA-associated mechanisms and the evidentiary standards needed to distinguish endogenous RNA responses, cargo association, and causal EV-mediated activity.

The preclinical literature, as a whole, supports a coherent but nuanced mechanistic model. EV/sEV therapies in cardiac injury most often reduce cardiomyocyte death, promote vascular recovery, restrain maladaptive remodeling, and reshape post-infarct inflammation through overlapping networks centered on AKT/ERK survival signaling, TGF-β/SMAD remodeling control, Notch-associated repair programs, and miRNA- or lncRNA-associated gene regulation [41,43,44,45,46,58,73,75]. Even so, most evidence remains preclinical, usually short-term, and often dependent on source-specific vesicle preparations that are not directly comparable across studies due to differences in producer cell state, purification, dose assignment, model systems, and delivery routes. Therefore, it is more reasonable to conclude that certain EV/sEV preparations can simultaneously modulate multiple injury response pathways rather than that EVs function as single target therapies.

## 5. Stem Cell-Derived EVs

As previously mentioned, EV populations, even from the same cell type, are heterogeneous. This is experimentally difficult and, in some cases, impossible to account for, so stem cell-derived EVs are best understood as a family of related but not interchangeable vesicles, interrelated by the producer cell type and environment. In this section, the studies are grouped by parent cell source because producer cell identity influences the therapeutic rationale, cargo profile, functional readout, and manufacturing strategy. MSC-derived EVs have the largest preclinical evidence base and are usually framed as cardioprotective, reparative, and immunomodulatory products. iPSC-derived EVs offer a renewable and more tunable cell source. Cardiac progenitor cell (CPC)-, cardiosphere-derived cell-, and cardiovascular progenitor-derived EVs are closely aligned with cardiac lineage programs, although these producer cell populations differ in origin, expansion history, and manufacturing feasibility [82,83,84,85].

Much of the rationale for the third pillar, myocardial regeneration, developed from earlier work on stem cells and stem cell-derived therapies. However, this history should not lead to describing all the benefits of stem cell-derived EV products as regenerative without stronger evidence of new, durable myocardial tissue formation.

### 5.1. From Cell Therapy to MSC-Derived EVs

The modern interest in stem cell-derived EVs arose largely from a major shift in cardiac cell therapy. Early strategies with bone marrow-derived cells, MSCs, CPCs, and c-kit+ cardiac cell populations were often discussed in terms of engraftment, transdifferentiation, or replacement of lost myocardium [86,87,88]. With time, however, that interpretation became less convincing. Donor cell persistence in the myocardium was usually limited, and rigorous lineage tracing studies challenged the idea that adult hematopoietic or c-kit+ cell populations generate enough new cardiomyocytes to explain functional recovery [89,90,91,92]. These studies did not eliminate the possibility that cell therapy could modify the injured heart, but they did shift the field away from a simple cell replacement model.

The remaining paradox was that functional improvement was still observed in some experimental and clinical settings, despite poor engraftment. This helped establish the paracrine hypothesis, in which transplanted cells act mainly by releasing soluble factors and vesicular signals that influence cardiomyocyte survival, inflammation, angiogenesis, and scar remodeling [93,94]. Later work further complicated the picture by showing that some benefits of cardiac cell therapy may arise from acute sterile immune activation and wound healing responses rather than direct regeneration [95]. Consensus discussions have, therefore, emphasized the need for more mechanistically defined approaches to cardiac repair, rather than reliance on poorly defined cell products [96,97].

EVs became important within this shift because they offered a plausible cell-free mediator of the stem cell secretome. They were no longer treated only as by-products of cultured cells, but as potential therapeutic agents capable of coordinating potential effects on recipient cardiac cells. Lai et al. [98] reported that MSC-derived sEVs reduced myocardial I/R injury, providing one of the earliest demonstrations that vesicles could reproduce part of the cardioprotective activity attributed to MSCs. Cardiosphere-derived EVs were then proposed as critical agents of cell therapy-induced cardiac regeneration; however, the reported endpoints more directly support cardioprotection and cardiac repair than confirmed myocardial regeneration. Related EV preparations were reported to reduce fibrosis, apoptosis, adverse remodeling, and functional decline after injury [83,84,99]. CPC-derived EVs were also shown to protect ischemic myocardium from acute I/R injury, indicating that cardiac lineage producer cells could release vesicles with direct therapeutic activity [82].

Within this family, MSC-derived EVs remain the most extensively studied stem cell-derived EV population in cardiac repair. This reflects the long history of MSCs in regenerative medicine and the practical advantages of MSCs as producer cells. MSCs can be isolated from bone marrow, adipose tissue, umbilical cord, placenta, dental pulp, and other tissues, and they are generally easier to expand than many lineage-restricted cardiac progenitor populations. The molecular complexity of MSC-derived EVs is part of their appeal, but it also makes potency assignment difficult when a study attributes benefit to one cargo molecule or pathway.

A preclinical systematic review of in vivo MI studies reported that MSC-derived EV treatment was generally associated with improved cardiac functional and histological outcomes [100]. Other reviews have reached similar conclusions, but also highlighted heterogeneity in MSC source, donor age, culture condition, isolation strategy, dose normalization, and outcome reporting [20,101,102,103,104]. Across preclinical studies, the most frequently reported endpoints include reduced cardiomyocyte death, increased angiogenesis, attenuated fibrosis, altered macrophage polarization, and improved ventricular function. These findings fit the paracrine model of MSC therapy, but place much of that activity into a smaller and more product-like vesicle fraction.

Several MSC EV studies went beyond describing benefit and have tried to link efficacy to cargo or parental cell conditioning. A miRNA sequencing study suggested that MSCs and MSC-derived sEVs may share related miRNA-associated mechanisms for enhancing cardiac repair [105]. The miR-21a-5p findings of Luther et al. [41] provide a prominent example of MSC sEV-associated cardioprotection. Related work reported that bone marrow MSC-derived sEV-associated miR-21 protected c-kit+ cardiac stem cells from oxidative injury through the PTEN/PI3K/AKT axis [106]. These studies support the idea that MSC-derived EV-associated miRNAs can be functionally important, while also emphasizing that the contribution of any individual cargo must be interpreted in the context of the full EV preparation and its producer cell state.

MSC EV activity can also be reshaped by the state of the producer cell. Hypoxia-elicited MSC-derived sEVs were associated with cardiac repair and miR-125b-dependent anti-apoptotic signaling after MI, and HIF-1α-overexpressing MSC-derived sEVs enhanced angiogenesis and cardioprotection in infarct models [57,107]. Other studies linked MSC-derived sEVs to immune modulation, including miR-24-3p-associated M2 macrophage polarization and miR-21-5p-associated regulation of inflammatory responses after reperfusion injury [108,109]. Together, these findings identify hypoxic, genetic, and inflammatory conditioning as source-specific variables that can alter MSC-EV activity.

### 5.2. iPSC-Derived EVs: Renewable and Lineage-Controlled Platforms

iPSC-derived EVs were developed from a different translational need. iPSCs and iPSC-derived cardiac cells provide a renewable platform for expansion, differentiation and control, which are, in principle, more systematic than primary cells. However, direct transplantation of pluripotent or incompletely differentiated cells raises concerns regarding tumorigenicity, undesirable differentiation and risk of arrhythmogenic events. EVs from iPSCs or iPSC-derived cardiac cells, therefore, became attractive as a way to capture part of the reparative secretome without transplanting proliferative cells.

In a preclinical comparison, Adamiak et al. [110] reported that iPSC-derived EVs produced stronger cardiac repair effects and fewer safety concerns than transplantation of the parent iPSCs in their MI model. Earlier work had also shown that EVs from iPSCs contained cardioprotective miRNAs and were associated with reduced cardiomyocyte apoptosis in ischemic myocardium [111]. EVs from iPSC-derived cardiomyocytes (iPSC-CMs) constitute a lineage-specific subgroup. In a murine acute MI model, Santoso et al. [112] administered iPSC-CM sEVs by intramyocardial injection and reported improved cardiac function and myocardial viability, reduced apoptosis and fibrosis, and enhanced autophagic activity. In a rat model of chronic ischemic cardiomyopathy, Tominaga et al. [113] administered clinical-grade iPSC-CM EVs intramyocardially two weeks after permanent left anterior descending (LAD) artery ligation; treatment improved left ventricle (LV) function and survival, increased angiogenesis and M2-like macrophage polarization, and reduced fibrosis and chronic inflammation. By contrast, Gao et al. [114] tested an EV preparation pooled from human iPSC-derived cardiomyocytes, ECs, and smooth muscle cells in a swine MI model. Intramyocardial administration improved functional, structural, and bioenergetic outcomes at four weeks without increasing arrhythmic events, but this mixed cardiac cell preparation should not be treated as equivalent to purified iPSC-CM EVs.

The activity of iPSC-derived EVs also depends on the differentiation state and culture conditions. Hypoxic culture promoted the angiogenic potential of human iPSC-derived EVs [31]. Hypoxia also enhanced the anti-fibrotic properties of human iPSC-derived EVs through a miR-302b-3p/TGF-β/SMAD2-related mechanism [115]. EVs secreted by ECs derived from human iPSCs improved recovery from MI in mice [113,116]. These findings support the possibility of source- and state-directed EV product design, with iPSC-EC-derived EVs better suited for vascular repair, iPSC-CM-derived EVs for myocardial stress responses, and hypoxia-conditioned EVs for stronger injury-adapted signaling, but head-to-head tests are still required to establish better therapeutic effects. Furthermore, this flexibility creates product definition challenges: the differentiated producer cell phenotype should be specified, and residual pluripotency-associated signals should be excluded from the final product.

### 5.3. CPC-, Cardiosphere-Derived Cell-, and Cardiovascular Progenitor-Derived EVs

CPC-, cardiosphere-derived cell-, and cardiovascular progenitor-derived EVs are considered together because their producer cells are all linked to cardiac lineage programs, although the categories remain biologically distinct. CPC-derived EVs have the advantage that their parental cells are more closely aligned with cardiac lineage programs than MSCs. The rationale is, therefore, somewhat different. MSC-derived EVs are usually framed as reparative and immunomodulatory, whereas CPC-derived EVs are often presented as more cardiac-related products for injured myocardium. Early work showed that CPC-derived EVs protected the ischemic myocardium from acute I/R injury [82]. Subsequent reviews and experimental studies have developed CPC-derived EVs as a cell-free therapeutic strategy for cardiac repair [85].

The evidence base is smaller than for MSC-derived EVs, but several studies support this source-specific rationale. CPC-derived EVs reduced infarct size and were associated with increased cardiovascular cell proliferation [117]. CPC-derived EVs were linked to EV-associated miR-21a-5p and PDCD4 repression and prevented cardiomyocyte apoptosis, showing that miRNA-mediated cardioprotection is not restricted to MSC-derived EVs [118]. At the same time, CPC-EV studies also illustrated major interpretive caution. Roefs et al. [119] discovered that EV-associated and co-isolated proteins in CPC-derived EV preparations stimulated angiogenesis. Even when an EV preparation is therapeutically active, the active component may not be a single RNA species and may not be entirely vesicular unless the isolation strategy and potency assay are designed to distinguish EV-associated activity from co-isolated soluble or protein complex material.

CPC-derived EVs have also been studied with translation in mind. A good manufacturing practice (GMP)-grade manufacturing approach for human CPC-derived EV preparations has been reported, which is an important advance for a product class that could, otherwise, remain limited to small laboratory scales [120]. CPC-derived EVs have also been modified to improve protection after MI, including miR-322 modification to enhance NADPH oxidase 2 (NOX2)-dependent angiogenic effects [121]. In addition, EVs from human embryonic stem cell (ESC)-derived cardiovascular progenitor cells promoted cardiac infarct healing by reducing cardiomyocyte death and promoting angiogenesis [55]. However, because the parental cells differ in origin, expansion history, lineage specificity, and manufacturing feasibility, EVs derived from cardiovascular progenitors, cardiosphere-derived cells, and CPCs will not necessarily have identical cargo or effects.

### 5.4. Critical Comparison of Stem Cell-Derived EV Sources and Emerging 3-Dimensional Platforms

Taken together, stem cell-derived EVs constitute a family of related but distinct therapeutic products. The source classes can be most usefully compared by the volume and type of available evidence, their translational rationale, and their principal sources of variability, rather than by pooling apparent efficacy across non-equivalent experiments. No source class can currently be ranked as uniformly superior. MSC-derived EVs have the deepest evidence base and the broadest range of reported actions, but they are also affected by donor tissue and age, passage, senescence, culture condition, priming strategy, and isolation heterogeneity [20,41,57,100,101,102,103,104,105,106,107,108,109]. Although their ultimate activity is largely dependent on differentiation state, culture protocol, and product characterization, iPSC-derived EVs offer a stronger case for renewable, potentially clonal production, donor control, and lineage engineering [31,110,111,112,113,114,115,116]. EVs from iPSC-derived cardiomyocytes and ECs may provide more lineage-directed signals, but their production is more complex and matched comparisons remain sparse. CPC-, cardiosphere-derived cell-, and cardiovascular progenitor-derived EVs have a more cardiac-aligned rationale and have shown anti-apoptotic, angiogenic, infarct reducing, and early manufacturing-related promise; unfortunately, producer cell definitions and the evidence base are smaller and less standardized [55,82,83,84,85,117,118,119,120,121]. The principal trade-offs among these and other relevant source classes are summarized in Table 1.

Direct matched comparisons remain rare. A recent head-to-head study compared sEVs from various cardiac-relevant cell sources, such as primary bone marrow MSCs, immortalized MSCs, ESCs, ESC-derived CPCs, ESC-derived cardiomyocytes, and ventricular cardiac fibroblasts, using common in vitro assays and same murine I/R model [122]. Within this matched experimental system, ESC-derived sEVs showed the most favorable angiogenic, anti-fibrotic, and remodeling profile among the tested products. This finding supports source dependence within the conditions examined, but it should not be generalized into a universal source ranking because the study evaluated a defined set of products, preparation conditions, doses, endpoints, and follow-up periods. Across the wider literature, apparent source effects remain confounded by media supplements, culture format, oxygen tension, isolation and purification, storage, administration dose, injury model, and potency assay. Therefore, rather than being merely a descriptive exercise, source comparison ought to be incorporated into the next phase of cardiac EV research.

Cardiac organoids and engineered 3-dimensional (3D) cardiac tissues represent emerging EV platforms rather than established therapeutic sources. Their multicellular architecture and extracellular matrix context may provide a more physiologically relevant setting for EV production and functional testing than conventional monocultures. Vital et al. [123] isolated EVs from human iPSC-derived cardiac organoids and found that 48.9% of the identified proteins overlapped with EVs from human heart explants, including proteins associated with metabolism and cardiac function. In a distinct use of 3D systems, Wagner et al. [124] added EC-derived EVs to engineered human cardiac tissues and observed improved vascular network length and continuity, supporting these tissues as EV-response and tissue-engineering platforms rather than as sources of the tested EVs. These studies support platform feasibility and physiological relevance, but they do not establish superior therapeutic potency of organoid-derived EVs over matched 2-dimensional cell-derived preparations. Translation will require reproducible control of organoid composition, scalable production, batch definition, and EV isolation from matrix-rich culture systems.

Overall, the current evidence supports indication- and product-specific source selection rather than a universal source ranking. The therapeutic activity of stem cell-derived EVs depends on the parent cell, culture condition, isolation workflow, and final EV preparation. This likely explains why different studies report overlapping but not identical outcomes. Cross-study comparisons should be considered descriptive unless the products are matched, while most existing studies establish the activity of a particular preparation relative to its own control rather than the superiority of one EV source class over another. It also connects directly to the translational issues for manufacturing, characterization, dose definition, and potency discussed in Section 9. The producer cell and state, EV identity, cargo profile, and potency readouts must be sufficiently detailed to know the identity of the product being tested before stem cell-derived EVs can be compared across studies or advanced toward clinical use. Accordingly, future comparisons should define EV products by producer cell identity, manufacturing workflow, cargo composition, and potency rather than treating stem cell-derived EVs as a single therapeutic class. Because many of the implicated pathways converge on post-transcriptional regulation, Section 6 examines regulatory RNA cargo in greater detail.

## 6. Regulatory RNA Cargo and Post-Transcriptional Gene Regulation

Evidence for EV-associated regulatory RNA is graded here according to the experimental chain demonstrated. Detection or enrichment establishes cargo association but not functional transfer. Uptake plus recipient cell target engagement supports pathway involvement, whereas a causal RNA-mediated mechanism requires appropriate loss-of-function evidence, such as donor cell depletion or recipient inhibition, ideally complemented by gain-of-function or rescue. Unless this chain was demonstrated, we describe the RNA as associated with or implicated in the reported effect rather than as its established mediator.

Our ability to sequence entire genomes and all of their transcribed RNAs has led to the discovery that only 1.2% of the human genome is used to encode proteins [125,126]. Those RNA transcripts that are not translated into protein are called ncRNAs [127,128]. At first, ncRNAs were considered to be junk, non-functional DNA sequences [129]. Nowadays, it is widely known that ncRNAs affect a wide range of cellular processes [130,131]. There are two types of ncRNAs: structural and regulatory RNAs. Transfer RNAs (tRNAs), ribosomal RNAs, small nuclear RNAs, and small nucleolar RNAs are structural ncRNAs that are crucial for cellular machinery [132]. Small ncRNAs of less than 200 nucleotides and lncRNAs of more than 200 nucleotides are the two primary classes of regulatory RNAs that govern gene expression [133,134,135]. Small ncRNAs include miRNAs, siRNAs, and PIWI-interacting RNAs (piRNAs) [133,134,135]. CircRNAs, which have different lengths and a covalently closed structure, are another important class of ncRNAs [130].

More than 800 miRNAs are expressed in the human heart, with miR-133a being one of the most abundantly expressed [136]. The focused subsections on miR-21, miR-126, the miR-133 family, and miR-210 were selected because these miRNAs recur in cardiac EV studies, represent complementary biological functions, including cardiomyocyte survival and fibroblast signaling, endothelial recovery and angiogenesis, cardiac lineage biology and remodeling, and hypoxia adaptation and mitochondrial stress, and have been examined through functional or mechanistic experiments. These examples are illustrative rather than an exhaustive catalog of cardiac EV-associated miRNAs.

Beyond their abundance in the heart, individual miRNAs have been implicated in endothelial dysfunction and cardiac remodeling. In human aortic, umbilical vein, and coronary artery endothelial cells, TNF-α reduced miR-27b-3p expression and induced inflammation, mitochondrial reactive oxygen species accumulation, loss of mitochondrial membrane polarization, cell cycle arrest, and apoptosis. Transfection with a miR-27b mimic attenuated these changes, partly through direct targeting of the FOXO1 3′-UTR and modulation of Akt/FOXO1 signaling, indicating a protective role for miR-27b-3p during inflammatory endothelial dysfunction [137]. However, this study used direct miRNA transfection and did not demonstrate EV-mediated delivery. In patients with heart failure undergoing cardiac resynchronization therapy, reverse left ventricular remodeling and improved myocardial performance were associated with increased circulating miR-26b-5p, miR-29a-3p, miR-30e-5p, miR-92a-3p, and miR-145-5p. These miRNAs have been linked to ionic-current regulation, fibrosis, apoptosis, and angiogenesis, respectively, although these clinical observations primarily establish association rather than direct causal involvement in remodeling [138].

### 6.1. Pathways of miRNA Biogenesis

The first step in canonical miRNA biogenesis is the transcription of miRNA genes in the nucleus by RNA polymerase II (RNAPII), which results in primary miRNA transcripts, which are long, capped, and polyadenylated miRNA precursors [137]. The RNase III enzymes, DROSHA and DICER, sequentially convert pri-miRNAs into mature miRNAs [137,138]. Pre-miRNA is a short hairpin of about 65 nucleotides with a 2-nucleotide 3′ overhang that is released when DROSHA cleaves the pri-miRNA [138]. The pre-miRNAs are then taken to the cytoplasm by exportin 5 (XPO5), a nuclear transport receptor [139]. Pre-miRNA is released into the cytosol when Ran guanosine triphosphatase is activated upon export, hydrolyzing guanosine triphosphate to guanosine diphosphate and initiating a complex disassembly. DICER removes the terminal loop of pre-miRNA in the cytoplasm [140,141,142], producing an Argonaute (AGO) protein-loaded 20 base pair RNA duplex with 2-nucleotide 3′ overhangs at both ends. One strand of the miRNA duplex is chosen as the functional guide strand while it is attached to AGO, and the other strand—the passenger strand—was assumed to be degraded because it was believed to be functionally inactive. After loading, the guide strand remains attached to AGO while the passenger strand is unwound and removed. The RNA-induced silencing complex (RISC), which is made up of mature miRNA bound to AGO, acts as the effector for gene silencing. This highly conserved canonical miRNA biogenesis pathway enables miRNAs to control post-transcriptional gene expression by binding to complementary sequences within the 3′ untranslated regions (UTRs) of target messenger RNAs (mRNAs), thereby promoting translational repression or mRNA degradation [143]. However, recent research has demonstrated that miRNAs may also interact with other regions, such as promoter sequences, coding regions, or 5′ UTRs, where they may increase the target gene’s expression in particular cellular contexts [144,145]. Noncanonical miRNA biogenesis pathways, which avoid DROSHA or DICER, are typically low in abundance and poorly conserved [146].

### 6.2. Sorting Mechanisms of miRNAs into sEVs

The miRNAs can be sorted into sEVs by a variety of processes, including the endosomal sorting complex required for transport (ESCRT) mechanism and several ESCRT-independent mechanisms. ESCRT-independent sorting mechanisms include those involving RNA binding proteins (RBPs), miRNA sequences, miRNA-induced silencing complex (miRISC), and neutral sphingomyelinase 2 (nSMase2). These mechanisms are context-specific. A change in extracellular RNA abundance does not, by itself, establish selective loading unless donor cell RNA abundance and EV production or release are appropriately controlled.

ESCRT is crucial for regulating MVB development. ESCRT-0 and inclusion proteins are known to enrich ubiquitinated cargo proteins and create microdomains on endosomal membranes. Cargo proteins were sorted into vesicles and vesicle formation was enhanced by ESCRT-I- and ESCRT-II-induced MVB vesicle budding. Ubiquitinated cargo proteins, including miRNA-protein complexes, are specifically sorted into exosomes during membrane budding and vesicle formation [147]. ESCRT-III shrinks and shears the bud neck to complete the membrane shedding process. Although miRNA–protein complexes have been proposed to intersect with this machinery, the cited evidence does not establish ESCRT-dependent loading as a general mechanism for miRNA incorporation into sEVs. Claims of selective miRNA loading, therefore, require cargo-specific perturbation of the proposed sorting pathway.

The majority of the cell’s RNA is found as ribonucleoprotein complexes [148]. By recognizing and binding to specific miRNA sequences, certain proteins have been shown to control miRNA sorting [149]. An essential ribonucleoprotein complex called Y-box binding protein (YBX1) can recognize and bind to a wide range of miRNAs. YBX1 depletion reduced miR-223 enrichment in sEVs, supporting a direct role for YBX1 in that experimental system [150]. Certain miRNA motifs were recognized and bound by YBX1, which facilitated their sorting into sEVs [151]. After YBX1 binds to the 5′ proximal sequence motif of miR-223, UCAGU, miR-223 is sorted into sEVs [152]. In addition, the ubiquitously expressed RBP, heterogeneous nuclear ribonucleoproteins A2/B1 (HNRNPA2B1), recognized and bound miR-198 via its short sequence motifs over-represented in miRNAs (EXOmotifs), regulating its loading into sEVs [153]. Other RBPs, like synaptotagmin-binding cytoplasmic RNA-interacting protein (SYNCRIP), bind to miRNA molecules with a short sequence motif to form a stable complex. Hepatocytes package this SYNCRIP-miRNA complex into sEVs, which are subsequently released into the extracellular space [154]. Wozniak et al. [155] found that a short sequence motif in miRNA cargo that binds to the RBP fragile X messenger ribonucleoprotein 1 (FMR1) and subsequently binds to the ESCRT machinery, was responsible for the enrichment of miRNA within sEVs. Teng et al. [156] found that the RBP, major vault protein (MVP), binds to the tumor suppressor, miR-193a, forming an MVP-miR-193a complex. This supports MVP-dependent export, not tumor growth inhibition caused by recipient cell exposure to an EV-delivered MVP–miR-193a complex.

Sorting sequences in miRNA influence their secretion in sEVs or retention in a cell. EXOmotifs are 4–7 nucleotide motifs that have been linked to miRNA enrichment in sEVs. CELLmotifs are 4–5 nucleotide motifs that have been linked to miRNA retention in cells [157]. These associations support sequence-dependent partitioning but do not alone demonstrate active loading or subsequent function in recipient cells. There is evidence that human cell-secreted sEVs contain mRNA fragments from the 3′ UTR, which contain miRNA target sites that direct the RISC to miRNA response elements on target transcripts [158]. However, this compositional finding does not establish recipient cell RISC engagement. According to Bolukbasi et al. [159], a zipcode-like 25-nucleotide sequence in the 3′-UTR was found in the majority of mRNAs enriched in sEVs. The miR-1289-mRNA complexes were sorted into sEVs after miR-1289 directly bound to the zipcode sequence. This supports a sequence-dependent sorting model, not functional transfer or gene regulation after uptake.

Proteins interact with mature miRNAs to form the miRISC. Argonaute 2 (AGO2), GW182, miRNA, and miRNA-repressed mRNA are components of miRISC. The AGO2-miRNA complex is created when AGO2 binds to miRNA. DICER and transactivation response TAR RNA binding protein (TRBP) are required for this process. TRBP recruits DICER to AGO2, promoting the loading of miRNA [160]. Base complementary pairing allows the AGO-miRNA complex to attach to target mRNA once it has formed [161]. The cleavage of target mRNAs is another crucial role of AGO2 in miRISC. Following the AGO-miRNA complex’s binding to target mRNAs, AGO2’s endonuclease activity is triggered, causing target mRNAs to be cleaved at particular locations and subsequently degraded [161]. Crucially, other proteins control AGO2’s role in miRISC. For instance, proteins like GW182 can interact with AGO2 to increase miRISC’s silencing activity [162].

Their interactions with membrane lipids have an impact on miRNA loading. RNAs constantly interact with the cytoplasmic surface of MVBs, and their affinity for lipid raft-like regions on the outer MVB membrane affects their incorporation into intraluminal vesicles [163]. nSMase is involved in MVB biogenesis and the conversion of sphingomyelin into ceramide in endosomal membranes. Increased miRNA expression in sEVs was observed when nSMase2 was overexpressed in oligodendrocytes and HEK cells. In contrast, a reduction in miRNA expression in sEVs was noted upon inhibition of nSMase2 [164,165]. Because nSMase also affects vesicle biogenesis, these experiments do not, by themselves, distinguish selective miRNA loading from changes in the number or release of vesicles. Examples of some of the well-characterized miRNAs are illustrated below.

### 6.3. miR-21

Encoded in the 17q23.2 locus of the human genome, miR-21 is a highly expressed and thoroughly researched miRNA [166]. It can be found in a variety of bodily fluids, is both extracellular and intracellular, and is linked to a number of molecular processes [166]. By controlling downstream target genes, miR-21 contributes to the pathophysiology of CVD. By controlling the expression of PTEN and PDCD4, miR-21 has anti-apoptotic effects [167,168]. The main target genes in cardiac fibrosis are sprouty 1 and 2 (SPRY1 and SPRY2) and SMAD7 [169,170,171]. Peroxisome proliferator-activated receptor alpha (PPARα), a-kinase anchoring protein 8 (AKAP8), BRCA1-associated RING domain 1 (BARD1), Jagged 1 (Jag1), sorbin and SH3 domain-containing protein 2 (SORBS2), and PDZ and LIM domain 5 (PDLIM5), are additional targets that have binding sites specific to miR-21 [172,173,174,175]. Overall, the emerging picture is that the function of miR-21 differs significantly depending on the cell type and the type of heart disease. In addition, miR-21-5p (i.e., the guide strand) and miR-21-3p (i.e., the passenger strand) have distinct, sometimes opposing functions within the different cardiac cell types.

By affecting processes like fibrosis, hypertrophy, ischemic injury, and cardiac stem cell proliferation, miR-21 contributes to cardiovascular pathophysiology. miR-21 inhibits SPRY1 in fibroblasts to regulate the ERK-mitogen-activated protein kinase (MAPK) signaling pathway, which in turn promotes fibroblast survival, growth factor secretion, and collagen synthesis [176]. After MI, the heart’s infarcted zones show a marked increase in miR-21, which promotes fibroblast differentiation into pathological myofibroblasts [177]. By inhibiting PTEN, miR-21 controls the expression of fibroblast metalloprotease-2 (MMP2) in fibroblasts of the infarct zone, which accelerates the development of fibrosis following I/R injury [178]. Furthermore, by stimulating the endothelial-to-mesenchymal transition, which is marked by the suppression of endothelial markers and the increase in fibroblast markers, upregulation of miR-21 in ECs promoted cardiac fibrosis [179]. In hypertensive conditions, elevated miR-21 levels are linked to improved cell survival, whereas miR-21 inhibition increases apoptosis [180]. miR-21 inhibits PTEN to increase cardiac stem cell viability [181]. In a rat model of I/R and a mouse model of MI, miR-21 had a protective effect on cardiomyocytes [182,183], which comprise 25–35% of all cells in the adult heart [184]. In line with these findings, overexpression of miR-21 reduced the size of infarcts by decreasing cardiomyocyte apoptosis [182]. Luther et al. [41] reported that MSC-derived sEVs were enriched in a miRNA cargo profile that included miR-21a-5p and were associated with reduced expression of apoptotic genes, smaller infarct size, and improved cardiac repair, with miR-21a-5p functionally implicated as an important contributor to the observed cardioprotection. Interestingly, Ramanujam et al. [185] reported that miR-21 was the highest expressed miRNA in cardiac macrophages both in health and disease. Mice with a macrophage-specific deletion of miR-21 were protected from pressure overload-induced interstitial fibrosis and cardiac dysfunction. In addition, miR-21 promoted a pro-inflammatory phenotype in cardiac macrophages that signal to and activate cardiac fibroblasts [185].

Many miRNA passenger strands that were previously thought to be subject to intracellular degradation, such as miR-21-3p, were found in sEVs derived from cardiac fibroblasts, according to Bang et al. [175]. Cardiac fibroblasts secreted sEVs containing miR-21-3p that were reported to be taken up by cardiomyocytes and associated with cardiomyocyte hypertrophy [175]. Additionally, inhibition of miR-21-3p reduced angiotensin II-induced cardiac hypertrophy [175]. In cardiac hypertrophy models, miR-21-3p acts as a protective factor by silencing histone deacetylase 8 (HDAC8) [186]. In human hearts, deep RNA sequencing revealed that miR-21-3p is expressed and elevated in heart failure [187]. Overexpression of miR-21-3p reduced pressure overload- and angiotensin II-induced hypertrophy, indicating a potential to prevent or reverse cardiac remodeling [186].

### 6.4. miR-126

The miR-126 gene is located within an intron on the epidermal growth factor-like-domain 7 (EGFL7) gene on chromosome 9 in humans [188,189]. Two mature miRNAs are produced by miR-126 biogenesis: miR-126-5p (i.e., the guide strand) and miR-126-3p (i.e., the passenger strand) [189]. ECs and endothelial progenitor cells are the main cells that express miR-126 [190]. Only the ECs of the kidney and heart express miR-126-3p [191].

miR-126 attenuates vascular inflammation, reduces EC apoptosis, and has pro-angiogenic and cardioprotective effects [192,193]. Transplantation of miR-126 overexpressing MSCs enhanced ischemic angiogenesis through AKT/ERK signaling in the infarcted myocardium [194]. During ischemic conditions, miR-126 promotes cardioprotection by activating pro-angiogenic and cell survival pathways [195]. miR-126 promotes angiogenesis in vascular ECs by activating the VEGF pathway [196]. MI patients had lower levels of serum miR-126-3p and plasma miR-126 than healthy people [197,198]. Interestingly, there is a positive correlation between the plasma concentration of cardiac troponin I, a marker of heart damage, and miR-126 [199]. Following miR-126 overexpression in adipose-derived stem cells (ADSCs), ADSC-derived sEVs showed increased miR-126 levels and were reported to increase the miR-126 signal in recipient cardiomyocytes. These findings were consistent with miR-126-associated protection, as sEV treatment reduced cardiomyocyte injury, promoted proliferation, and attenuated apoptosis. Furthermore, by blocking the pro-inflammatory factors interleukin-1β (IL-1β), IL-6, and tumor necrosis factor-α (TNF-α), sEVs containing miR-126 from ADSCs reduced inflammation in vitro [200]. Endothelial progenitor cell-derived EVs stimulated cardiac fibroblast proliferation and angiogenic behavior, increased endothelial markers, and reduced fibrosis-associated proteins and HMGB1 [201]. By stimulating cardiac fibroblasts, upregulating the expression of EC-specific markers, and downregulating fibrosis-associated proteins, endothelial progenitor cell-derived sEVs containing miR-126 produced a cardioprotective effect [202]. High levels of miR-126-3p and miR-126-5p are found in endothelial progenitor cell-derived sEVs [203], while hydrogel-delivered endothelial progenitor cell-derived EVs improved angiogenesis and cardiac function after MI [204]. In human umbilical vein ECs undergoing endothelial-to-mesenchymal transition (EndMT), Jordan et al. [191] found that miR-126-3p was downregulated; overexpression of miR-126-3p prevented EndMT.

### 6.5. miR-133

miR-133a-1, miR-133a-2, and miR-133b are miR-133 genes found on human chromosomes 18, 20, and 6, respectively. Notably, the final two nucleotides at the 3′ terminus of miR-133b differ from those of miR-133a and miR-133a-2. According to Ohanian and Humphreys [205], the three miRNAs are transcribed as bicistronic transcripts with miR-1-2 and miR-133a-1, miR-1-1 and miR-133a-2, or miR-206 and miR-133b, respectively. Both cardiac and skeletal muscle express the bicistronic miR-1/miR-133a cluster [206,207]. The transcription of miR-1 and miR-133a is regulated by a number of important myocyte differentiation factors, including myocyte enhancer factor 2 (MEF2), serum response factor (SRF), myogenic differentiation-1 (MYOD1), and myocardin (MYOCD) [208]. Cardiomyocytes and cardiac fibroblasts are the primary cells that express miR-133 [209].

The role of miR-133a in cardiac development and function is well-established [210]. Additionally, according to Shao and Zhang [211], miR-133a is a crucial regulator of cardiomyocyte differentiation, proliferation, and other processes necessary for the development of the heart. Mice with double knockout of both miR-133a loci showed increased apoptosis and excessive cardiomyocyte proliferation linked to increased expression of their targets, cyclin D2 and SRF, which resulted in ventricular septal deficits [212]. In addition to severe myocardial fibrosis and eventual heart failure, rats lacking the miR-133a gene display aberrant cardiac structural development, including ventricular septal defects, enlarged cardiac chambers, and thinned ventricular walls [213,214,215,216]. Overexpression of miR-133a in postnatal cardiomyocytes [217] or in muscles [218] has no effect.

The aberrant expression of miR-133a has been linked to heart hypertrophy [219], cardiac fibrosis [220], and heart failure [221,222]. In mice, rats, and humans, transaortic constriction-induced hypertrophy downregulates miR-133a [223,224]. miR-133a knockdown (KD) was sufficient to induce cardiac hypertrophy [223]. Transgenic expression of miR-133a improved diastolic function and decreased myocardial fibrosis without affecting the degree of hypertrophy in pressure-overloaded adult hearts [217]. Overexpression of miR-133a in cardiomyocytes attenuated cardiomyocyte hypertrophy and pyroptosis, effects mediated by translational repression of IκB kinase ε (IKKε) by miR-133a-3p [225]. In the failing heart, only twelve miRNAs exhibited differential expression. One of the downregulated miRNAs in failing hearts was miR-133a [226]. An analysis of surgical samples of the right atrial myocardium of patients showed that miR-133 expression decreased significantly as the severity of heart failure increased [227]. According to Yang et al. [228], ischemia preconditioning-induced serum sEVs suppressed replacement fibrosis to promote cardiac repair post-MI. miR-133a-3p was implicated in the cardioprotective effects of ischemic preconditioning-induced sEVs in ischemic myocardium. In that study, miR-133a-3p–associated protection was linked to repression of latent TGF-β-binding protein (LTBP1) and protein phosphatase 2, catalytic subunit, alpha isozyme (PPP2CA) and the suppression of the TGF-β pathway. In addition, miR-133a-3p-loaded sEVs facilitated myocardial repair post-MI in rats [229]. In rats with acute MI, Cao et al. [230] found that miR-133a from transplanted miR-133a-transfected BMSCs reduced cardiac fibrosis by preventing cardiac fibroblasts from differentiating into myofibroblasts and decreasing collagen deposition. Chen et al. [231] found that the cardiac-specific expression of miR-133a decreased cardiac fibrosis. Lastly, miR-133a prevented fibrosis by modulating several components of the beta-adrenergic receptor signaling pathway [232].

### 6.6. miR-210

miR-210 is a hypoxia-inducible miRNA. It is induced in a variety of primary and transformed cells under hypoxic conditions [233]. An intron of a noncoding RNA that is transcribed from AK123583 (MIR210HG) on chromosome 11p15.5 contains the stem-loop of miR-210 [234]. HIF-1α and HIF-2α regulate miR-210 [234,235]. The hypoxia-responsive element (HRE) on the proximal miR-210 promoter is bound by HIF-1α [236]. The functional HRE is ~40 base pairs upstream of the predicted transcription start site, suggesting that this HRE is responsible for the induction of AK123483 [234].

Direct miR-210 gain- and loss-of-function studies, separate from EV delivery experiments, showed that miR-210 improves heart function by inhibiting apoptosis [237,238], stimulating angiogenesis [237], supporting cardiomyocyte proliferation [239,240], and promoting a shift from mitochondrial respiration to glycolysis [241]. By directly suppressing the protein expression of E2F transcription factor 3 (E2F3), miR-210 prevented oxygen-glucose deprivation/reperfusion-induced cardiomyocyte apoptosis [242]. In cardiomyocytes under oxidative stress, overexpression of miR-210 decreased the generation of ROS, thereby preventing apoptosis [243,244]. In cardiomyocytes under hypoxic stress, overexpression of miR-210 reduced oxidative stress and apoptosis; inhibition of miR-210 increased oxidative stress and apoptosis [245]. Furthermore, miR-210 overexpression inhibited the known pro-apoptotic proteins caspase 8-associated protein 2 (CASP8AP2) and apoptosis-inducing factor mitochondrion-associated 3 (AIFM3) [243,246]. It was shown that miR-210 can prevent apoptosis by altering cardiomyocyte metabolism. By promoting an alteration of energy metabolism, miR-210 inhibited the expression and activity of the iron–sulfur cluster assembly enzyme (ISCU) and sirtuin 3 (SIRT3) mRNA and protein levels under oxidative stress, thereby indirectly preventing cardiomyocyte apoptosis [247]. Additionally, miR-210 inhibited myocardial apoptosis by downregulating the expression level of the pro-apoptotic protein, BCL-2 adenovirus E1b 19kDa-interacting protein 3 (BNIP3) [248]. Wang et al. found that MSC EVs enriched in miR-210 increased EC proliferation, migration, and tube formation and enhanced angiogenesis in vivo; reducing miR-210 in the producer MSCs impaired these effects, while ephrin A3 (EFNA3) repression supported recipient cell target engagement [249]. Interestingly, miR-210 overexpression in normoxic ECs promoted the development of structures resembling capillaries [244]. In a rat MI model, miR-210 was injected into the myocardium. VEGF expression doubled and the vascular density in the vicinity of the infarct area doubled four weeks later, suggesting an improvement in angiogenesis [240]. Furthermore, Bei et al. [239] found that miR-210 targeted cyclin-dependent kinase 10 (CDK10) and EFNA3 to promote cardiomyocyte survival and proliferation in stem cell-derived cardiomyocytes and a human cardiomyocyte cell line. Finally, miR-210 directly targets the anti-proliferation gene adenomatous polyposis coli (APC). APC was downregulated by overexpression of miR-210, which increased cardiomyocyte proliferation [240].

### 6.7. Pathway of LncRNA Biogenesis

Like mRNAs, RNAPII transcribes the majority of lncRNAs in the nucleus, producing transcripts with 3′-end poly(A) tails and 5′-end 7-methylguanosine caps [133]. Other RNA polymerases do, however, transcribe some lncRNAs [133]. Due to ineffective splicing, sequence motifs, and interactions with RBPs, many RNAPII-transcribed lncRNAs are inefficiently processed [250,251] and are kept in the nucleus [252,253]. Nuclear RNA export factor 1 (NXF1) exports other lncRNAs via nuclear pore complexes to the cytoplasm, where they interact with RBPs, bind to ribosomes, localize to organelles, or disperse throughout the cytoplasm [254]. Roughly half of the cytoplasmic mRNA-like lncRNAs are attached to ribosomes. This enables them to suppress translation by competing with mRNAs for mRNA entry sites in the ribosomes [255]. Small open reading frames that ribosomes engage for the possible translation of micropeptides are present in ~30% of the highly expressed mRNA-like lncRNAs [256]. Furthermore, a large number of lncRNAs have binding sites for proteins or miRNAs, which sequester these molecules and may alter cytoplasmic function, signaling pathways, and miRNA stability [133,257].

### 6.8. Packaging of LncRNA into sEVs

RBP-dependent lncRNA loading has been demonstrated principally in noncardiac cancer models. Several RBPs recognize and bind to a wide range of miRNAs, allowing them to be packaged into sEVs [150,153,156]. Likewise, RBPs bind to lncRNAs, allowing them to be packaged into sEVs. The lncRNA called lymph node metastasis-associated transcript 2 was loaded into sEVs by the RBP HNRNPA2B1 [258]. HNRNPA2B1 played a role in loading the lncRNA, lncARSR, into sEVs [259]. Heterogeneous nuclear ribonuclear protein A1 (HNRNPA1), after sumoylation, recognizes the lncRNA, ELNAT1, thus mediating its packaging into sEVs [260]. These findings support context-specific RBP-dependent loading rather than a universal lncRNA sorting mechanism.

### 6.9. Gene Regulation by LncRNAs

By interacting with DNA, RNA, or proteins, lncRNAs are essential components of gene regulatory networks [261,262]. As a result, lncRNAs are involved in many physiological and pathological processes, such as cardiovascular disorders [263,264,265]. Du et al. [263] found that the lncRNA, the dilated cardiomyopathy repressive transcript (DCRT), was highly enriched in heart tissues. Patients with dilated cardiomyopathy had reduced expression of it in their hearts. Contractile dysfunction in cardiomyocytes is caused by insufficient lncRNA DCRT, which disrupts its interaction with polypyrimidine tract binding protein 1 (PTBP1) in the nucleus. This results in increased splicing of NADH dehydrogenase ubiquinone oxidoreductase core subunit 2 (NDUFS2) and an imbalance in mitochondrial homeostasis [263]. Apart from controlling alternative splicing [263], lncRNAs also control gene expression through a process called “sponging” [266], which involves directly binding miRNAs and preventing their interaction with their target mRNA. For example, the target genes of miR-296, integral membrane protein 2a and tumor protein p53-inducible nuclear protein 1 (TP53INP1), were derepressed by the lncRNA cardiac regeneration-related long noncoding ribonucleic acid (CAREL), which sponged miR-296. CAREL overexpression reduced cardiomyocyte proliferation and division indices, whereas CAREL silencing increased these indices and improved cardiac function after MI, findings consistent with a pro-regenerative response [266]. The lncRNA known as myosin heavy chain-associated RNA transcripts (MHRT) has cardioprotective properties [267]. Overexpression of MHRT activated nuclear factor erythroid 2-related factor 2 (NRF2) to inhibit angiotensin II-induced activation of the NLRP3 inflammasome, ROS accumulation, and oxidative damage in cardiomyocytes [268]. Furthermore, the lncRNAs NONHSAG005537 and NONHSAG017620 prevented human cardiac fibroblasts from proliferating, migrating, invading, and transforming in response to TGF-β. Therefore, in cardiac fibrosis, these lncRNAs had a cardioprotective function [269]. The lncRNA, the myocardial infarction-associated transcript (MIAT), is a significant contributor to cardiac damage. It is upregulated in ischemic conditions and exacerbates myocardial injury [270]. Myocardial fibrosis was successfully treated by lowering MIAT [271], and myocardial injury was lessened by silencing MIAT [272]. According to Ma et al. [273], myocardial cells from rats and humans exposed to hypoxia/reoxygenation (H/R) injury were more viable when MIAT was silenced. Important pathological processes, such as oxidative stress, myocardial dysfunction, and calcium (Ca^2+^) overload, were reduced by MIAT silencing. MIAT silencing improved the histological characteristics of heart tissue, stabilized cardiac function, and decreased infarct size by ~49% in a rat MI model [273].

### 6.10. Pathway of CircRNA Biogenesis

Precursor mRNAs are synthesized by RNAPII and spliced into linear mRNAs. Unlike linear mRNAs, circRNAs are formed through a process called back-splicing (tail-to-head circularization) [274]. Back-splicing takes place when both ends of a single pre-mRNA molecule connect covalently to form a closed circle, joining the 3′ splice site to the 5′ splice site, forming a “head-to-tail” circular molecule [275]. Their circular shape makes circRNAs highly stable and resistant to exonuclease degradation [276] as they lack 5′ caps and 3′ poly(A) tails [277].

### 6.11. Packaging of CircRNA into sEVs

circRNAs were found to be enriched in sEVs despite being downregulated in the parental cells, according to several studies [278,279,280]. This supports the possibility that circRNAs may be selectively enriched into sEVs. Despite the fact that the mechanism of circRNA loading into sEVs is still largely unknown, Zang et al. [281] found that fused in sarcoma (FUS) binds to hypoxia-related circRNAs through its zinc-finger Ran-binding protein (Zf_RanBP) domain and is implicated in sEV loading in neurons.

### 6.12. Sponge Functions of CircRNA

circRNAs are widely expressed in the cardiovascular system [282,283] and make up a significant portion of the mammalian transcriptome [284]. Heart failure and MI are linked to abnormal circRNA expression [285,286]. By directly binding miRNAs and blocking their interaction with their target mRNA—a process known as “sponging”—circRNAs control gene expression [287].

Circular RNA CDR1 antisense (circ-Cdr1as) provides a related example linking circRNA sponge activity to macrophage phenotype and post-MI repair. In bone marrow-derived macrophages, circ-Cdr1as was downregulated in pro-inflammatory cells and upregulated in anti-inflammatory cells; its overexpression promoted anti-inflammatory markers, whereas KD had the opposite effect [288]. In a later MI study, cardiac circ-Cdr1as overexpression or injection of circ-Cdr1as-overexpressing macrophages increased reparative macrophage markers, reduced infarct size, and improved left ventricular function. Mechanistic experiments supported a circ-Cdr1as/miR-7/Krüppel-like factor 4 (KLF4) axis [289]. These studies did not evaluate circ-Cdr1as loading into sEVs or functional EV-mediated transfer, but they illustrate how circRNA sponge mechanisms can influence inflammatory resolution after cardiac injury.

One of the most highly expressed circRNAs in the mouse heart is the tetratricopeptide repeat domain 3 (circ-TTC3). Circ-TTC3 was shown to directly bind miR-15b by Cai et al. [290]. The sequestration or sponging of miR-15b by circ-TTC3 protected cardiomyocytes against ischemia-induced apoptosis [290]. Heart-related circRNA (HRCR) was shown to directly bind to miR-223 in the cytoplasm of cardiomyocytes [291]. HRCR functioned as an endogenous sponge to sequester and suppress the activity of miR-223, thereby preventing heart failure and cardiac hypertrophy [291]. In addition, in rat MI hearts, quercetin reduced the size of the infarct and enhanced heart function. The cardioprotective effects of quercetin were mediated by downregulation of circPAN3, which sponges miR-221 [292]. Chen et al. [293] found that circCBFB sponges miR-495-3p. CircCBFB expression was upregulated, and miR-495-3p expression was downregulated in cardiomyocytes in response to H/R. By reducing apoptosis, oxidative stress, and mitochondrial dysfunction via miR-495-3p, the lack of circCBFB had a cardioprotective effect against H/R-induced cardiomyocyte injury [293]. Lastly, the downregulation of circTRRAP, a miRNA sponge for miR-214-3p, mediated the protective effects of salvianolic acid B on cardiomyocytes exposed to I/R. Thus, circTRRAP silencing had cardioprotective effects by disinhibiting miR-214-3p [294].

### 6.13. Potential Post-Transcriptional Gene Regulation After sEV Uptake

After sEV uptake, regulatory RNAs can influence recipient cells mainly through post-transcriptional control of gene expression. For miRNAs, this usually involves binding to complementary or partially complementary regions in target mRNAs, most often in the 3′ UTR, followed by translational repression, mRNA destabilization, or both. This mechanism is well-suited for cardiac injury because the relevant outcomes, including apoptosis, oxidative stress, endothelial repair, autophagy, inflammation, and fibrosis, are controlled by regulatory networks rather than by one single gene.

Several cardiac miRNAs, including miR-21, miR-126, miR-133a, and miR-210, have been characterized herein. One additional example is bone marrow MSC-derived sEV-associated miR-29c, which reduced myocardial I/R injury by inhibiting excessive autophagy through the PTEN/AKT/mechanistic target of rapamycin (mTOR) pathway [295].

Longer ncRNAs further extend this post-transcriptional network. In hypoxia/reoxygenation-injured H9C2 cells, bone marrow MSC-derived sEVs were reported to alleviate injury through the heart and neural crest derivatives expressed transcript 2-antisense RNA 1 (HAND2-AS1)/miR-17-5p/mitofusin 2 (MFN2) axis, improving cell viability while reducing apoptosis, oxidative stress, and inflammatory injury [296]. This lncRNA-miRNA-mRNA relationship shows that sEV effects may be indirect. A vesicle-associated or vesicle-induced lncRNA may sponge a miRNA and, thereby, release a protein-coding mRNA from repression. Therefore, the functional cargo may not always be the RNA molecule most abundant in the vesicle.

Hsp70.3 is a good cardiac stress response example of the post-transcriptional regulation complexity that goes beyond miRNA binding. Hsp70.3 expression is controlled by both miRNAs and alternative polyadenylation. Shortening of the Hsp70.3 3′ UTR can remove a suppressive miR-378a-5p binding site and permit stronger protein induction during ischemic or heat shock stress [297]. Although not a study of EVs, it shows that cardiac stress genes are regulated through 3′ UTR length, miRNA accessibility, RNA binding context, and transcript stability, the same regulatory architecture that sEV-associated miRNAs may engage after uptake.

### 6.14. Selected EV-Associated Cardiac RNA Examples

Specific sEV-associated RNA examples illustrate how the post-transcriptional mechanisms outlined above may contribute to cardiac outcome. Apart from miR-21a-5p [41] and miR-221 [42], research also suggests that recipient cell phenotype and cardiac outcome are influenced by miR-132-3p [61] and lncRNA-associated signaling [46]. For example, lncRNA HCP5 in human bone marrow MSC-derived sEVs alleviated myocardial I/R injury by sponging miR-497 and activating insulin-like growth factor 1 (IGF1)/PI3K/AKT signaling [46]. Previous research using EVs/microvesicles derived from iPSCs also reported increased cardioprotective miRNA signals and decreased cardiomyocyte apoptosis in ischemic myocardium [111]. Together, these studies show how RNA-associated cargo may intersect with survival, immune, angiogenic, and remodeling phenotypes without constituting a separate therapeutic pillar.

### 6.15. Evidence Standards for EV-Associated RNA Claims

The detection of candidate cargo in sEVs should be interpreted with caution. Stronger mechanistic evidence requires vesicle uptake, cargo transfer, target engagement, and loss- or gain-of-function experiments showing that blocking the cargo changes the phenotype. Increased miRNA signal in recipient cells after EV treatment can reflect true transfer, EV adherence to the cell surface, uptake into non-functional compartments, or induction of endogenous miRNA expression after EV-triggered signaling. Studies based mainly on cargo enrichment, recipient cell quantitative reverse transcription polymerase chain reaction (qRT-PCR), pathway activation, or inhibitor effects should, therefore, be interpreted as cargo-associated or pathway-consistent unless they establish the required causal chain. This is especially important in cardiac sEV therapy, where multiple vesicular and producer cell factors may converge on the same survival and repair pathways.

## 7. Non-RNA Cargo: Proteins, Lipids, Organelles, and Metabolic Repair

### 7.1. Protein and Lipid Co-Cargo as Stress Response Regulators

Although regulatory RNA is the main focus of Section 6, sEV activity is not carried by RNA alone. sEVs also contain proteins and lipids that reflect the condition of the producer cell and influence vesicle formation, uptake, and downstream signaling. This is particularly relevant in cardiac injury, where hypoxia, oxidative stress, metabolic stress, and inflammation all affect producer cell state before vesicle release.

For example, cardiac-specific Hsp20 overexpression in diabetic cardiomyocytes increased sEV generation through interaction with TSG101, and the released vesicles contained higher levels of Hsp20, phosphorylated AKT, survivin (BIRC5), and superoxide dismutase 1 (SOD1). These vesicles protected against hyperglycemia-induced cell death and improved diabetic cardiac remodeling, suggesting that stress response proteins can be both cargo and regulators of vesicle biogenesis or activity [38]. Plasma sEVs from healthy donors have also been shown to protect the myocardium from I/R injury through a Hsp70/TLR4-dependent pathway, while remote ischemic conditioning-derived plasma sEVs improved post-MI function and angiogenesis, with Hsp70 implicated [60,298]. Circulating EVs collected after exercise similarly showed enrichment of antioxidant activity in hydrogen peroxide-stressed human iPSC-derived cardiomyocytes [299]. These findings suggest that heat shock proteins and antioxidant proteins can act as stress-adaptive signals in selected settings.

Protein cargo may also interact with RNA cargo. In sepsis-induced cardiomyopathy, endothelial Hsp family A member 12B (HSPA12B) was linked to the regulation of miR-126 expression and serum sEV-associated miR-126 levels; sEV-associated miR-126 also reduced adhesion molecule expression, inflammatory cell infiltration, and cardiac dysfunction [300]. This suggests that the stress response protein is not independent from the RNA mechanism and helps define the vesicle message.

Lipid cargo and lipid-dependent vesicle formation add another layer. Hypoxic MSC-derived sEVs had increased miR-210 and stronger reparative effects after MI, and inhibition of nSMase2 reduced miR-210 secretion and weakened protection [301]. This connects lipid-dependent EV biogenesis and regulatory RNA export, even though lipid cargo is not the therapeutic component. Lipids may also act as functional cargo. Vascular endothelial cell-derived sEVs carrying sphingosylphosphorylcholine attenuated myocardial I/R injury through nuclear receptor 4A2 (NR4A2)/optineurin (OPTN)-mediated mitophagy and reduced cardiomyocyte apoptosis [302]. Thus, protein and lipid cargo should be considered part of the same therapeutic unit as regulatory RNA, especially when producer cells are stressed before vesicle collection.

### 7.2. Autophagy and Metabolic Adaptation as Convergent Response Pathways

Autophagy and metabolic adaptation appear to be convergent EV response pathways, but not all studies establish a defined regulatory RNA cargo. Adipose-derived MSC sEVs protected against I/R injury through Wnt/β-catenin signaling [303], Danshen-pretreated bone marrow MSC sEVs promoted anti-apoptosis together with increased autophagy [304], and umbilical cord MSC sEVs regulated autophagy in diabetic cardiomyopathy and viral myocarditis through 5′ AMP-activated protein kinase (AMPK)-centered pathways [305,306]. Similar autophagy-associated benefits have been observed for iPSC-cardiomyocyte sEVs [112], bone marrow MSC-derived sEVs acting through AMPK/peroxisome proliferator-activated receptor gamma coactivator-1α (PGC-1α) signaling [307], and intrapericardial sEV therapy linked to forkhead box O3 (FOXO3) activation [308]. Consistent with these findings, EVs from different tissues produced overlapping cardioprotective effects while retaining source-specific differences [309]. These findings imply that autophagy and metabolic adaptation are recurrent stress response programs through which different EV preparations may limit injury, although the operative cargo and proximal mechanism vary among models. They should, therefore, be viewed as convergent modulators of cellular stress responses rather than as a separate therapeutic pathway.

### 7.3. Metabolic Stress and Inter-Organ EV Signaling

Most therapeutic discussions surrounding EV therapy in the heart have focused on regulatory RNAs, proteins, and lipids as commonly discussed candidate mediators of cardioprotection and repair. That remains appropriate, especially for the miRNA- and lncRNA-centered studies discussed above. However, the preceding protein and lipid cargo examples, together with the autophagy and metabolic adaptation studies, establish why non-RNA cargo should be considered together with organelle-associated material and metabolic stress signaling. An emerging body of work suggests that vesicle-mediated benefit may also extend into organelle-associated cargo and metabolic support. This area should still be considered developing rather than fully established. Although the literature increasingly suggests that EVs can influence mitochondrial homeostasis, redox signaling, and metabolic adaptation, some of the most informative studies in this area extend beyond strictly canonical sEVs and include mixed EV populations, mitochondria-enriched vesicles, or biomimetic nanovesicles [23,310]. Therefore, rather than being an equally developed branch of cardiac EV/sEV biology, this body of research is best understood as a developing mechanistic frontier. Even so, it substantially expands the therapeutic framework of EV research and represents a meaningful conceptual shift for the field. According to this more comprehensive perspective, vesicles may deliver mitochondrial material or regulators of mitochondrial quality control, Ca^2+^ handling, adenosine triphosphate (ATP) production, and oxidative stress responses, thereby reshaping the metabolic competence of injured myocardium in addition to transmitting instructive signaling cues [23,310].

One of the clearest examples of this shift comes from natural metabolic crosstalk between adipose tissue and the heart under stress. Crewe et al. [311] discovered that EVs, including a sEV-enriched fraction containing respiration-competent but oxidatively damaged mitochondrial particles, are quickly released by energetically stressed adipocytes. After entering the bloodstream and being taken up by cardiomyocytes, these vesicles caused a transient rise in ROS, which in turn triggered the heart’s compensatory antioxidant pathways [311]. Importantly, rather than producing net injury, this stress signal appeared to function in a preconditioning-like manner, and administration of adipocyte-derived EVs, particularly a sEV-enriched fraction, reduced I/R injury in vivo [311]. Loyer et al. [312] later emphasized the broader significance of this discovery by characterizing it as a type of inter-organ mitohormesis, in which the myocardium is conditioned to better withstand subsequent oxidative damage by adipose tissue under energetic stress. This adipocyte-to-heart axis should be viewed as a particularly illuminating model rather than as a rule for all adipose EVs or all metabolic stress. This work supports the idea that oxidatively damaged mitochondrial cargo in adipocyte-derived EVs can occasionally act as a protective signal rather than just a cellular waste product [311,312].

The interpretation of donor cell state in EV biology is also altered by these findings. In the past, metabolic stress in EV-producing cells has frequently been viewed primarily as a confounding factor that changes vesicle yield or composition in an unpredictable way. Instead, the aforementioned studies on adipocytes point to a different possibility: the metabolic state of the producer cell may encode biologically significant information that influences vesicle function in ways that are adaptive [311,312]. Additional adipose-linked studies support this general idea, although not all are equally central to the mechanistic argument. For example, Chai et al. [313] reported that early cold water treatment combined with adipose-derived MSC sEVs protected rat hearts from I/R injury, with better left ventricular ejection fraction (LVEF) and lower oxidative stress, inflammatory, mitochondrial damage, and fibrosis-associated markers. Likewise, brown adipocyte β3-adrenergic receptor signaling has been associated with cardioprotection through suppression of EV-associated inducible nitric oxide synthase (iNOS), again supporting the view that adipose-derived EVs can influence the redox and inflammatory setting of the injured myocardium in a metabolically relevant manner [314]. All of these findings point to the possibility that producer cell metabolic status is more than just an annoyance in experiments, but rather a useful indicator of EV function.

### 7.4. Cardiac Evidence for Mitochondrial Rescue by EV Cargo

The more established signaling-based paradigms of EV biology are not replaced by emerging organelle-cargo mechanisms; rather, they probably coexist [23,310]. This is an important conceptual point. It would oversimplify biology to draw a false distinction between “signaling cargo” and “metabolic cargo.” Instead, EV-mediated cardioprotection may operate on several levels simultaneously: some cargoes primarily reprogram transcriptional, inflammatory, or survival pathways, whereas others may support mitochondrial function, ATP generation, Ca^2+^ homeostasis, or oxidative stress adaptation [23,310]. This integrated perspective is especially relevant in cardiac injury, where early bioenergetic collapse, ROS excess, mitochondrial dysfunction, and cell death are tightly coupled events. Additionally, it was discovered that EVs/sEVs prevented stress-induced damage to dystrophin-deficient cardiomyocytes by lowering ROS, delaying the opening of the mitochondrial permeability transition pore (MPTP), maintaining mitochondrial membrane potential, and activating ERK1/2/p38 mitogen-activated kinase (p38MAPK) signaling in a way that depended on EV/sEV surface proteins [43]. These results imply that vesicle treatment can start at least some early metabolic benefits at the membrane level, rather than requiring direct delivery of internal organelle cargo [43]. As a result, the organelle-cargo model should be seen as an extension of RNA- or protein-centered paradigms rather than their replacement.

Cardiac and ischemia-related studies beyond the adipocyte field provide further support for this broader model of mitochondrial rescue. In a non-cardiac but mechanistically relevant ischemic model, Dave et al. [315] showed that mitochondria-containing EVs increased ATP production in recipient brain ECs and decreased infarct size in mice, supporting the possibility that organelle-bearing vesicles can contribute to bioenergetic rescue across ischemic tissues. Liu et al. [316] reported that cardiac-derived EVs protected against I/R injury by delivering mitochondrial ATP synthase F1 subunit alpha (ATP5A1) and improving mitochondrial function, providing a myocardial-specific example of metabolically active vesicle cargo. Zhou et al. [317] further showed that PTEN-induced kinase 1 (PINK1) contained in human MSC-derived sEVs prevented mitochondrial Ca^2+^ overload in septic cardiomyocytes by restoring mitochondrial Ca^2+^ efflux, thereby extending the relevance of mitochondrial homeostasis beyond ischemia. Similarly, O’Brien et al. [318] found that mitochondria-rich EVs rescued patient-specific cardiomyocytes from doxorubicin injury, improving contractility, ATP production, mitochondrial biogenesis, and survival. Notably, a large EV preparation rather than a sEV population was the most effective fraction [318]. That is not an unimportant detail. It emphasizes how some of the most convincing organelle-transfer discoveries in a cardiovascular context do not fit neatly into the strictest definition of sEV.

### 7.5. Mechanistic and Translational Uncertainties

Because of this, the mechanistically unresolved issues are just as significant as the promising ones. The field still faces substantial uncertainty regarding vesicle subclass identity, cargo integrity, uptake mechanism, intracellular processing, and the exact therapeutic entity responsible for benefit [23,310]. Some studies appear to transfer intact or partially intact mitochondrial material, whereas others may primarily deliver mitochondrial proteins, mitochondrial DNA, or regulators of mitochondrial quality control that secondarily restore bioenergetics [23,310,316,317,318]. These possibilities could all lead to improved ATP production or lower ROS, but they are not the same therapy. They would differ in potency, duration of effect, biodistribution, storage stability, immunogenicity, and the type of release testing needed for translation. These questions are further complicated by the heterogeneity of many published EV preparations, making it difficult to determine whether the relevant therapeutic entity is a true sEV, a larger EV, or a mixed population [23,310,315,318]. Thus, although organelle-cargo transfer is mechanistically compelling, mitochondrial rescue should be described as an emerging mechanism, since it is not yet an established category of sEV biology.

### 7.6. Engineered Mitochondria-Focused EV Platforms

Despite these drawbacks, engineered mitochondria-focused platforms are already starting to shape the field’s translational direction. Instead of focusing on naturally occurring metabolic rescue, several studies have attempted to engineer vesicle-based therapeutics that deliberately improve mitochondrial recovery. Melatonin-loaded adipose-derived biomimetic nanovesicles improved myocardial repair after MI and were associated with lower apoptosis, lower ROS generation, more microvessel formation, less fibrosis, and improved mitochondrial function [319]. Yang et al. [320] described engineered MSC-derived sEVs for dual delivery of SIRT3 and glycosylphosphatidylinositol-insulin to improve uptake, mitochondrial function, oxidative stress, glucose metabolism, infarct size, and cardiac function in rat I/R models.

Other studies extend mitochondrial rescue into toxic, septic, and ferroptotic injury settings. Trophoblast stem cell-derived sEVs alleviated doxorubicin cardiotoxicity, in part, by improving mitofusin-2 (MFN2)-mediated mitochondrial fusion [321]. IL-1β-stimulated macrophage-derived sEVs were reported to improve septic myocardial injury through miR-146a-dependent regulation of inflammatory signaling and mitochondrial homeostasis via the MAPK4/dynamin-related protein 1 (DRP1) pathway [322]. Furthermore, GATA-binding protein 4 (GATA4)-overexpressing BMSC-derived sEVs inhibited H/R-induced ferroptosis through regulation of the BRCA1-associated protein 1 (BAP1)/solute carrier family member 11 (SLC7A11)/inositol 1,3,4,5-tetrakisphosphate receptor (IP4R) axis, with MPTP opening and mitochondrial dysfunction, suggesting that mitochondria-centered vesicle therapy may also interact with regulated cell death pathways [323]. Together, these findings indicate that mitochondrial rescue is increasingly being investigated as a programmable therapeutic goal rather than just a naturally occurring characteristic of donor-derived EVs. Rather than depending exclusively on endogenous paracrine cargo, the next stage of EV therapy may involve intentional tuning of mitochondrial quality control, fusion-fission balance, Ca^2+^ handling, and oxidative stress.

### 7.7. Synthesis: Mitochondrial Rescue as an Emerging Therapeutic Axis

Across the studies summarized above, mitochondrial homeostasis emerges as a convergence point for multiple EV-associated mechanisms, including redox control, metabolic adaptation, mitophagy, and—under more restrictive experimental conditions—organelle transfer. Collectively, these mechanisms may modify how recipient cells, including cardiomyocytes, handle ROS, Ca^2+^, ATP generation, and mitochondrial quality control [23,310,311,316,317,318,319,320,321,322,323]. These mechanisms should be distinguished experimentally: improved respiration, ATP production, or mitochondrial function does not demonstrate transfer of intact mitochondria alone, because such effects may result from mitochondrial proteins, regulatory RNA, lipids, surface-mediated signaling, or other indirect responses. An sEV-enriched fraction released by energetically stressed adipocytes contained respiration-competent but oxidatively damaged mitochondrial particles, and reduced I/R injury is especially important because it reframes metabolic stress from being merely injurious to being, in the right vesicular context, preconditioning [311]. All of these results point to a significant conceptual shift in the field. However, compared to the more established RNA-, protein-, and immune-centered mechanisms covered elsewhere in this review, this is still a developing field. It is valuable because it shows that the metabolic state of donor cells can influence vesicle function in ways that are biologically significant. It also opens a translational path whereby EVs are designed to carry instructive signals and restore mitochondrial competence in tissues that have been exposed to ischemic, septic, or toxic injury [23,310,311,313,314,315,316,317,318,319,320,321,322,323].

## 8. Cardiac Injury Models and Outcomes

This section examines where therapeutic EVs and sEVs have been tested and what types of cardiac benefit have been measured, rather than focusing on how they function at the pathway level. MI models produced by permanent coronary ligation or related ischemic injury are currently the most popular experimental platforms in the field. There is a smaller but growing body of work in I/R, chemotherapy-associated cardiotoxicity, hypertrophic remodeling, viral myocarditis, diabetic injury, and cardiac organoid systems [11,324,325,326,327,328,329,330,331,332,333,334,335,336]. Across these preclinical studies, therapeutic EV/sEV administration has frequently been associated with improved LVEF and fractional shortening (FS), smaller infarct or scar burden, less ventricular dilation, and lower histologic or molecular evidence of tissue injury and inflammation [11,324,325,326,327,328,329,330,331,332,333,334,335,336]. However, these findings establish context-specific activity of particular preparations and the outcome is not a universal property of the EV class itself.

Direct comparison across studies remains limited because producer cell identity and state, isolation and purification, product characterization, dose normalization, administration route and timing, injury model and species, endpoint definition, and follow-up duration can each alter apparent efficacy [22,101,103,104,337,338,339,340,341,342,343,344,345,346,347,348,349,350,351,352,353]. Table 2 should, therefore, be read as a context-specific evidence map rather than as a ranking of EV sources or delivery formats. The largest concentration of outcome studies remain concentrated in rodent MI and I/R models, whereas non-ischemic indications are expanding but remain less mature. Delivery enhancing techniques like spray administration, targeting peptides, surface engineering, and hydrogel anchoring remain less extensively replicated. These outcomes provide important evidence of cardioprotection and cardiac repair, whereas regeneration requires additional evidence of durable new myocardium and functional integration.

### 8.1. Permanent Ligation and Acute MI Models

The most heavily represented setting remains AMI, especially rodent left anterior descending (LAD) coronary artery ligation models [113,116,117,354,355,356,357,358,359,360,361,362,363,364,365,366,367]. These models are useful because they generate relatively reproducible endpoints. In these studies, efficacy is usually assessed by echocardiography, infarct or scar quantification, ventricular geometry, fibrosis burden, and cell survival within the injured myocardium. LVEF and FS are the most common functional readouts, but they are not sufficient on their own; the most convincing studies are those that pair functional improvement with histology, infarct burden, ventricular remodeling indices, and cell-specific injury markers [18,39,59,66,110,113,116,117,122,316,336,354,355,356,357,358,359,360,361,362,363,364,365,366,367,368,369,370,371,372,373,374,375,376,377,378,379,380,381,382,383,384,387]. Together, these measures provide a more complete picture of functional and structural repair, whereas evidence of regeneration requires a separate demonstration of durable new myocardial tissue and functional integration. EVs or sEVs derived from CPCs, MSCs, endothelial lineage cells, placental or endometrial MSCs, pericardial adipose tissue, and iPSC-derived cardiomyocytes have generally been associated with improved systolic performance and reduced infarct expansion, fibrosis, apoptosis, or adverse remodeling after MI [116,117,354,355,356,357,358,359,360,361,362,363]. More recent studies extend these findings to chronic ischemic cardiomyopathy, pericardial adipose tissue EVs, minimally invasive spray delivery of EVs/sEVs, peptide-assisted EV retention, and progenitor cell vesicles in which deleterious cargo has been depleted, again with improvement in ventricular function and structural repair endpoints [113,364,365,366,367].

Importantly, the outcome differences between studies that employ vesicle sources are not insignificant. Matched comparisons indicate that EV source or product form can influence outcome. González-King et al. [122] compared several sEV sources under common experimental conditions and found the most favorable repair profile for ESC-derived sEVs within that test system. By contrast, Adamiak et al. [110] compared iPSC-derived EVs with the parent iPSCs—not with other EV source classes—and reported greater repair and fewer safety concerns with the EV preparation in that model. These findings support product-specific effects but do not justify ranking EV source classes across the wider setting. This observation is important because it argues against treating EVs derived from different stem cell and progenitor cell sources as functionally equivalent. They also do not establish a universal source hierarchy because few studies hold EV preparation, dose, model, and outcome assessment constant. Instead, the MI literature increasingly supports the view that EV producer cell biology is itself an outcome-determining variable. Relatedly, post-infarct remodeling after the initial ischemic event has been attenuated by repeated remote ischemic conditioning and by strategies designed to improve myocardial homing, including ischemic myocardium-targeting peptides and surface engineering of systemically delivered vesicles [18,66,368]. In this respect, the MI literature has become informative for demonstrating efficacy and identifying which product and delivery features are most likely to matter in practice. However, the translational evidence hierarchy is still biased toward small animal proof-of-concept rather than large animal validation studies because the majority of the directly cited efficacy studies are still rodent-based [11,18,66,110,113,116,117,122,324,325,326,327,328,329,330,331,332,333,334,335,336,354,355,356,357,359,360,361,362,363,364,365,366,367,368,369,370,371,372,373,374,375,376,377,378,379,380,381,382,383,384,387].

### 8.2. I/R Models

A second major preclinical platform is I/R injury, which is particularly informative because it captures both the ischemic damage and the additional damage imposed by reperfusion, including oxidative stress, microvascular dysfunction, inflammatory cell recruitment, and mitochondrial damage [59,316,369,370,371,372,373,374]. In these studies, EVs/sEVs from anoxia-preconditioned MSCs, cardiomyocytes, plasma, and other cardiac or circulatory sources, as well as platelet membrane-fused or hydrogel-anchored formulations, have been associated with smaller infarcts, preservation of systolic performance, reduced microvascular obstruction, and less inflammatory cell infiltration after reperfusion [59,316,369,370,371,372,373,374]. Many of these studies also go beyond LVEF and evaluate reperfusion-specific phenotypes such as endothelial recovery, no-reflow or microvascular obstruction, neutrophil trafficking, neutrophil extracellular trap formation, oxidative injury, and mitochondrial functional indices, making the I/R model especially useful when comparing vesicle products with matched experimental design across studies [39,59,316,369,370,371,372,373,374]. Thus, the I/R models frequently offer a clearer picture of whether a particular vesicle preparation can reduce acute reperfusion-associated injury and preserve tissue that is still salvageable, even though permanent ligation MI models are essential for documenting broad structural and functional repair.

However, there is still some heterogeneity within the I/R models. Some studies administer EVs before injury, some at reperfusion or soon afterward, and others during a later repair phase. These designs test different therapeutic ideas and should not be combined. A pretreatment protocol is closer to cardioprotection or preconditioning, while post-I/R dosing is more relevant to clinically realistic rescue therapy. Also, at least one cardiomyocyte-derived sEV study is better described as an endogenous or treatment-induced signaling study rather than as an exogenous in vivo EV administration [59].

### 8.3. Non-Ischemic Animal Models, In Vitro Systems, and Organoids

Beyond ischemic infarction, a growing set of studies uses non-ischemic cardiac injury models to test whether the beneficial outcome profile generalizes beyond coronary occlusion [375,376,377,378,379,387]. In chemotherapy-associated injury, sEVs from CPCs, MSCs, adipose-derived MSCs, and trophoblast-related sources have been reported to preserve cardiac function and reduce fibrosis, oxidative stress, apoptosis, inflammation, or adverse remodeling in doxorubicin-, doxorubicin/trastuzumab-, or cyclophosphamide–induced dilated cardiomyopathy models [375,376,377,378,379,387]. These studies provide insight into EV benefit in cardiac injury that is not primarily caused by coronary occlusion, but the number of models, dosing schedules, and outcome measures is smaller and more varied.

Other recent work extends outcome testing to radiation-induced cardiac organoid injury treated with umbilical cord MSC-derived sEVs, cardiac hypertrophy treated with targeted NADPH oxidase 4 (NOX4) siRNA-loaded sEVs, aged diabetic cardiomyocyte injury treated with VCAM-1 high umbilical cord MSC-derived sEVs, and viral myocarditis treated with either an engineered dual-targeting sEV vaccine or platelet-derived EVs, showing that vesicle-based benefit can also be captured in organoid, hypertrophic, metabolic, and inflammatory models [380,381,382,383,384]. In this setting, cardiac organoids function as recipient and functional testing models; their emerging use as EV-producing platforms is discussed in Section 5.4. These studies are important not only because they broaden the possible indication space, but also because they introduce endpoint families that are not well captured in standard infarct models, including organoid viability and Ca^2+^ handling, hypertrophy indices, ferroptosis-associated injury, and immune cell-linked inflammatory damage [380,381,382,383,384]. At present, however, these non-ischemic models remain less mature than the MI and I/R models and should be interpreted as an expansion of therapeutic scope rather than evidence of equal depth across all cardiac indications.

### 8.4. Cross-Model Heterogeneity and Context Dependence

However, the outcome literature is not always positive, and this variation is instructive in and of itself. Just because a vesicle population is small or naturally derived does not mean that it is reparative. Vesicles from M1-like macrophages can worsen post-infarct dysfunction, and P8 myocardial tissue EVs were reported to induce a macrophage phenotype that suppressed neonatal cardiomyocyte proliferation in co-culture, indicating that not every EV population is reparative simply because it is small or naturally derived [62,385]. By contrast, M2 macrophage-derived vesicles and MI plasma-derived EVs have been associated with improved angiogenesis, better function, or more favorable post-AMI remodeling [61,73,79,386], while anti-fibrotic benefit has also been documented in selected remodeling models [67]. This is one reason why direct source comparisons remain important, and why reviews and meta-analyses continue to emphasize careful attention to dose, timing, and outcome reporting [22,110,122,336,347,348,349,350,351,352,353]. It also underscores why this section should not be read as a simple catalog of favorable results. Instead, one ought to conclude that the benefit is contingent upon the relationship between the vesicle source, the injury setting, the administration route, and the endpoints selected to define success.

### 8.5. Route, Retention, and Formulation Effects

Route of administration and formulation can also influence measured outcomes. Intramyocardial injection, intravenous delivery, pericardial or epicardial administration, spray administration, and hydrogel-assisted local retention have all been studied [18,22,66,347,348,349,350,351,352,353,365,373,374]. These design choices should be treated as study variables, because reviews and meta-analyses of biomaterial-assisted delivery support the idea that retention and release kinetics can change the apparent therapeutic effect [22,347,351,352,353]. Therefore, interpretation of cardiac outcome data should not separate EV biology from delivery design; in many cases, the therapeutic signal reflects a combined product: vesicle source and cargo, route, formulation, local retention, and the biology of the injury model.

Taken together, the preclinical evidence supports a relatively consistent phenotype-level conclusion. In permanent ligation MI, I/R injury, and several non-ischemic cardiac injury settings, therapeutic EV/sEV formulations most often improve LVEF and FS, reduce infarct or scar size, and mitigate apoptosis- and inflammation-associated injury readouts. The most compelling studies, however, are those that combine these overall results with ventricular remodeling metrics and tissue-level histology, as they demonstrate that the benefit is a more comprehensive change in post-injury repair rather than just a temporary imaging effect [18,39,59,66,110,113,116,117,122,316,336,354,355,356,357,358,359,360,361,362,363,364,365,366,367,368,369,370,371,372,373,374,375,376,377,378,379,380,381,382,383,384,387]. The recurrent alignment of these outcome patterns with lower cell death, lower inflammatory burden, improved vascular recovery, and reduced maladaptive remodeling is consistent with the mechanisms nested within the cardioprotection and cardiac repair pillars discussed in Section 4, but the key point here is more straightforward. Across multiple cardiac injury models, EV/sEV therapy is repeatedly measurable at the level of function, structure, and tissue damage, which is why these models remain central to the evidence base for cardiac translation [22,61,73,316,347,348,349,350,351,352,353,385,386]. The next translational question is whether these model-specific benefits can be converted into a defined, reproducible therapeutic product.

## 9. Challenges and Translation Barriers

**Figure 2:**
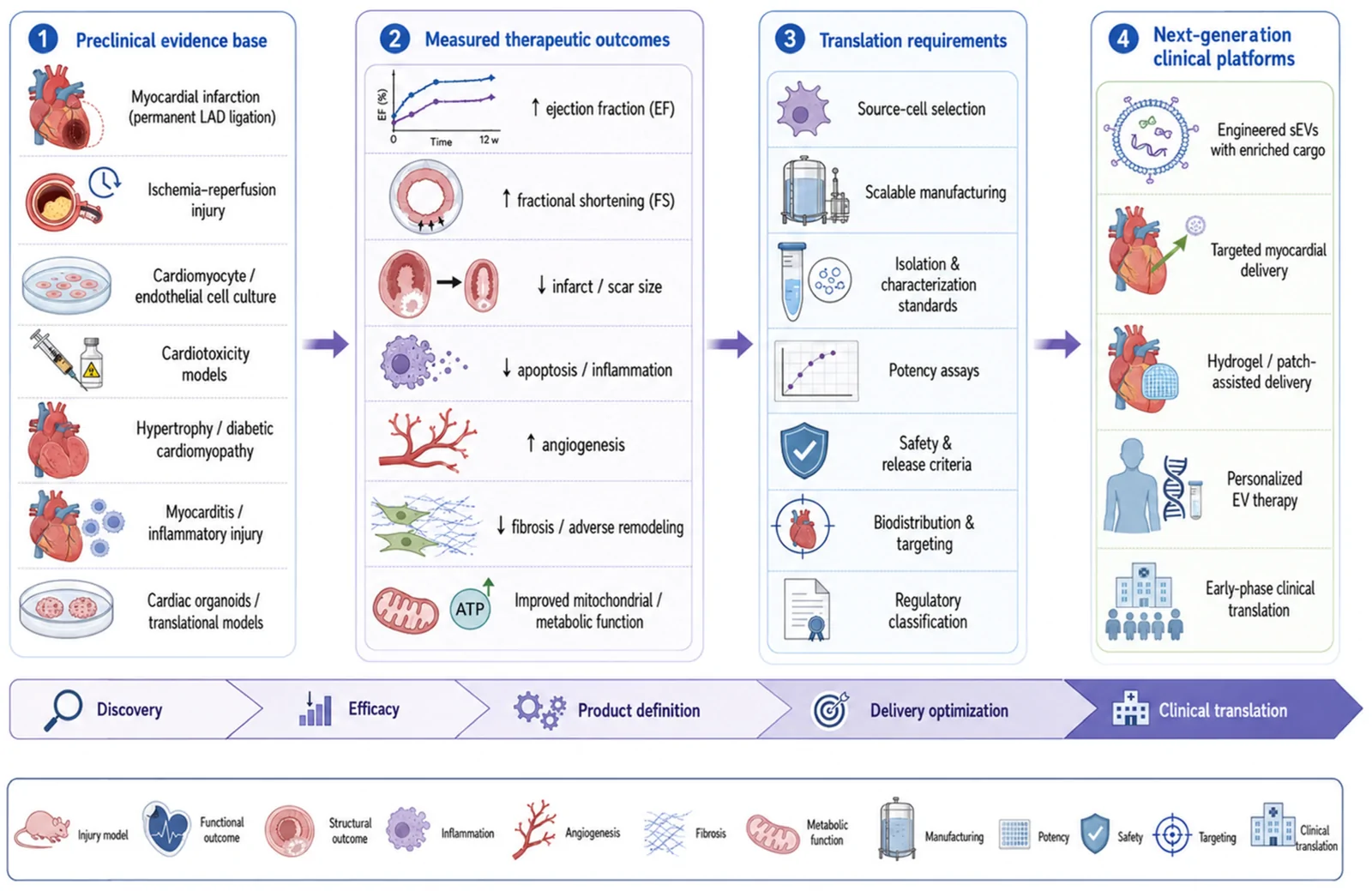
From preclinical evidence to clinical translation of cardiac sEV therapy. The preclinical evidence base includes myocardial infarction (permanent LAD artery ligation), ischemia–reperfusion injury, cardiomyocyte/EC culture, cardiotoxicity models, hypertrophy/diabetic cardiomyopathy, myocarditis/inflammatory injury, and cardiac organoids/translational models. Measured therapeutic outcomes include increased ejection fraction, increased fractional shortening, reduced infarct/scar size, reduced apoptosis/inflammation, increased angiogenesis, reduced fibrosis/adverse remodeling, and improved mitochondria/metabolic function. Translation requirements include source cell selection, scalable manufacturing, isolation and characterization standards, potency assays, safety and release criteria, biodistribution and targeting, and regulatory classification. Next-generation clinical platforms include engineered sEVs with enriched cargo, targeted myocardial delivery, hydrogel/patch-assisted delivery, personalized EV therapy, and early-phase clinical studies. The stages of therapeutic development are discovery, efficacy, product definition, delivery optimization, and clinical translation. Abbreviations: LAD, left anterior descending; sEVs, small extracellular vesicles. This figure was created with BioRender.com. http://app.biorender.com/ (accessed on 28 June 2026).

The central translational question in cardiac EV research is becoming less about whether these EVs/sEVs are bioactive. Many preclinical studies show that they are. The more difficult question is whether they can be produced as a consistent and scalable therapeutic product. Preclinical studies increasingly support the view that EV-based preparations can reproduce many of the reparative effects once attributed primarily to cell therapy, including cell survival, immunomodulation, and support of vascular and myocardial recovery (see Figure 2). Yet biological activity alone is not sufficient for clinical development. In cardiac repair, efficacy claims are often demonstrated using small, experimentally optimized batches produced under tightly controlled laboratory conditions, whereas clinical development demands batch-to-batch reproducibility, stable formulation, validated release criteria, and a delivery strategy capable of placing sufficient product at the site of injury [24,350,388]. Thus, the main barrier is not the absence of biological promise, but the difficulty of converting a heterogeneous secreted product into a medicinal product that can be manufactured, tested, stored, dosed, and regulated.

### 9.1. Manufacturing Scale and Product Heterogeneity

The scalability of EV production is the first significant obstacle (see Figure 2). Source cell identity, culture conditions, media composition, passage number, and bioprocess design are all closely related to EV yield and composition. These factors can change not only the number of vesicles recovered but also their composition and functional output [120,350,388]. This is not merely a technical manufacturing variable. Rather, it reflects the underlying biology of EV biogenesis. Even within broadly similar stromal or CPC products, substantial heterogeneity can emerge from donor source, ischemic conditioning, and pharmacologic preconditioning. For example, ischemia altered the heterogeneity and EV ribosomal protein profile of epicardial adipose tissue-derived stem cells, suggesting that the stress state of the producer cell can become part of the final vesicle phenotype [389]. Likewise, sEVs released from Suxiao Jiuxin pill-treated cardiac MSCs differed functionally from untreated ones, showing that pharmacologic preconditioning can reshape EV-mediated recipient cell effects [390]. Similar concerns arise when xeno-free adaptation or other media changes are introduced to improve clinical manufacturability, because such changes may enhance regulatory acceptability while simultaneously altering reparative potency [391]. Therefore, “same cell type” and “same EV product” are not always synonymous.

Donor-to-donor variability is also substantial, especially for primary MSC-, CPC-, adipose-, or blood-derived EV products. Even when the nominal cell source is the same, donor age, sex, metabolic state, CVD, diabetes, inflammation, medication exposure, and other comorbidities may alter EV yield, miRNA and protein cargo, lipid composition, metabolite content, and biological activity. As a result, EVs derived from different donors may not be functionally equivalent even if they show similar particle size, surface marker enrichment, and total protein profiles. This is a major challenge for donor qualification, master cell bank selection, potency testing, comparability, and regulatory review. Therefore, future cardiac EV products will likely need either tightly controlled donor selection or renewable producer cell platforms that reduce donor-dependent variability.

Simultaneously, the field has been pushed toward manufacturing innovation by the pressure to reach therapeutic scale. According to reports of GMP-oriented production of human CPC EVs/sEVs, quality control, downstream purification, and controlled upstream expansion are theoretically possible [120]. Other approaches, including scalable generation of human iPSC-derived nanovesicles and bioelectronic stimulation to increase EV production, also move the field toward higher output platforms [392,393]. These approaches move the field beyond proof-of-concept biology toward process-oriented product development, but they also expose a second translational problem. Strategies that increase output may result in EVs or EV-like particles that are not exactly the same as naturally secreted endosomal sEVs. The membrane composition, cargo loading, and uptake behavior of mechanically produced nanovesicles, stimulus-enhanced EV preparations, and highly conditioned source cell products may be different from those of more native sEV populations [350,388,392,393]. As a result, gains in manufacturability may come at the cost of added comparability questions, especially when the final product differs in membrane composition, cargo loading, or uptake behavior. Scalability and product definition are inextricably linked in clinical translation.

### 9.2. Isolation, Characterization, and Critical Quality Attributes

A related barrier is the lack of standardization of isolation, purification, and characterization (see Figure 2). Cardiac EV studies still use a mixture of differential ultracentrifugation, precipitation, filtration, SEC, tangential flow filtration, or hybrid workflows. Each of these choices affects particle recovery, co-isolated proteins or lipoproteins, and the apparent cargo profile of the preparation [24,350]. Section 3 explains why isolation, purification, and characterization affect biological interpretation. In translation, the same issues become critical quality attributes because a cardiac EV product must be reproducibly identifiable and comparable across lots.

MISEV2023 has helped the field move away from overconfident labeling of all small particles as “exosomes” and has established a more rigorous framework for nomenclature, pre-analytical variables, separation methods, and characterization [24]. However, product variability is not eliminated by better reporting standards. For translation, the key question is not only whether a preparation meets minimal characterization criteria, but whether the manufacturing workflow yields a product with stable critical quality attributes across lots, over time, and potentially across sites. In practice, this means that particle counts and a small panel of positive markers are unlikely to be sufficient. Translationally credible release specifications will likely need to integrate identity, purity, potency, stability, and appropriate safety testing, while also accounting for the possibility of residual impurities introduced during culture or isolation [120,350,388]. In other words, characterization has to move from “Does this look like an EV preparation?” toward “Is this the same product as the previous lot, and does it retain the intended biological activity?”.

### 9.3. Safety and Release Criteria

Safety and release criteria are still underdeveloped for cardiac EV therapy (see Figure 2), but they cannot be added only at the end. For a cardiac EV product, release cannot realistically be reduced to nanoparticle tracking data and a small positive marker panel. A clinically credible product will also need evidence addressing sterility, endotoxin or pyrogen burden, residual protein or lipoprotein contamination, storage stability, freeze–thaw robustness, and the possibility of off-target accumulation after systemic dosing [350,388]. Product biodistribution and off-target accumulation are addressed separately in Section 9.5.

Existing preclinical safety studies provide an important starting point. Human umbilical cord MSC-derived sEVs were reported to be well tolerated in animal models, with no adverse effects in hemolysis, vascular and muscle stimulation, systemic anaphylaxis, pyrogen testing, or standard liver and renal safety readouts after intravenous administration [394]. Similarly, sEVs derived from umbilical cord blood showed no significant in vitro or in vivo toxicity after repeated high dose intravenous administration, but their biodistribution was mainly in clearance organs such as the liver, spleen, and kidneys rather than in specific therapeutic target tissue [395]. These findings are reassuring, but they do not establish safety for every cardiac EV product, especially engineered products, drug-loaded vesicles, or repeated dose regimens. They also reinforce that safety evaluation for EV therapeutics must address not only acute tolerability, but also biodistribution, repeated dose exposure, formulation stability, and lot release criteria that can be implemented reproducibly during manufacturing [350,388,394,395].

### 9.4. Potency Testing and Dose Normalization

The issue of potency testing is equally significant and still unsolved (see Figure 2). For lot release, an EV product should demonstrate biological function linked to the intended mechanism of action and be suitable for lot release [396]. In cardiac repair, this is particularly difficult because EVs can act through multiple overlapping pathways, including cell survival, immunomodulation, angiogenesis, anti-fibrotic signaling, and metabolic support. A single generic assay is, therefore, unlikely to fully capture the full spectrum of therapeutically relevant activity.

This challenge becomes clear when comparing different cardiac applications. MSC secretions improved donor heart function after ex vivo cold storage, and related secretome preparations preserved myocardial transcriptomic programs during prolonged cold ischemia, suggesting that preservation biology may depend on secretome composition, potentially including both vesicular and soluble factors and is sensitive to storage conditions [397,398]. By contrast, bone marrow MSC-derived sEVs alleviated immune checkpoint inhibitor-associated myocardial injury through macrophage polarization and pyroptosis-related mechanisms, pointing to a distinct immunomodulatory potency axis [46]. Similarly, human cardiac EVs released under various inflammatory conditions supported angiogenesis in various ways, again underlining that function is highly dependent on the state of the progenitor cells and the biological context [63]. Taken together, these studies are encouraging, but they also indicate that potency testing in cardiac EV development may need to be indication-specific, mechanism-informed, and compatible with routine lot release, rather than reduced to a universal angiogenesis assay or particle-dose metric [396].

Dose normalization remains a related unresolved issue (see Figure 2). Studies dose EVs by particle number, protein amount, producer cell equivalent, EV-associated cargo, or total preparation volume [350,388]. These dosing approaches are not interchangeable. Protein-based dosing can be distorted by contaminants, particle counts do not measure functional cargo, and producer cell equivalents depend heavily on culture conditions. Studies should, therefore, report the dose per administration, number of administrations, cumulative dose, preparation concentration and volume, normalization basis, and body weight adjustment when applicable. Until dose reporting becomes more consistent, it will remain difficult to compare studies or define a rational starting dose for clinical testing.

### 9.5. Biodistribution and Targeting Efficiency

Another significant obstacle is biodistribution and targeted delivery (see Figure 2). One recurrent lesson from cardiac EV studies is that the injured myocardium is not automatically the dominant destination of a systemically administered EV product. The route of administration, particle dose, vesicle source, surface composition, and disease state all influence where the therapeutic ultimately localizes and which cell types receive it [350,399]. Studies evaluating CPC-derived EV distribution indicate that tissue and cellular biodistribution are measurable, potentially manipulable, and highly relevant to therapeutic interpretation [399]. But they also show a practical limitation: after intravenous injection, much of the EV signal may accumulate in the liver rather than in the heart.

This has stimulated interest in engineering strategies to improve tropism, including membrane cloaking, surface display, and other targeting modifications [400]. Cardiomyocyte targeting EV/sEVs from sulforaphane-treated fibroblasts have been tested in infarcted rats, reflecting the effort to make cardiac delivery less dependent on passive biodistribution [401]. Targeted delivery, however, does not completely resolve the translational issue. Surface modifications and other engineering steps add to the complexity of production and may change the identity, comparability, and regulatory classification of the product [350,388,400,401]. Targeted delivery may, therefore, reduce one obstacle while also making others more difficult to overcome.

### 9.6. Regulatory Classification

Regulatory classification remains unsettled (see Figure 2). EV therapeutics do not fit neatly into a single historical framework, as their classification depends on the source cells, manufacturing steps, level of handling, cargo engineering, and intended mode of action [350,388]. A relatively native stromal cell-derived sEV preparation is not regulatorily equivalent to a membrane-engineered targeted vesicle, a drug-loaded EV, or a mechanically generated nanovesicle. This distinction has practical consequences. It complicates the chemistry, production and control expectations, raises questions about the qualification of raw materials and the variability of donors, and makes comparative studies particularly important when process changes are introduced during development [120,350,388].

In this context, GMP compliance is necessary, but may not be sufficient on its own. A product can be made under GMP conditions and still lack a clear potency assay, stable release criteria, or an adequate comparability strategy. For cardiac EV therapy, upstream cell banking, culture expansion, harvest timing, purification, storage, and final formulation all need to be connected to the final biological activity. Otherwise, process changes could produce a preparation that looks similar by particle count and surface markers but behaves differently in vivo.

Overall, the translational bottleneck for cardiac EV therapy lies in the convergence of these issues. Manufacturing scale without compositional control is not sufficient for successful translation. Targeting without a stable product definition may improve biodistribution but weaken comparability. Standardized characterization without mechanism-linked potency testing is unlikely to support lot release. For the field to advance, future studies will need to align manufacturing strategy, analytical characterization, biodistribution profiling, and potency testing around the intended clinical indication from the outset [24,350,388,396]. In that sense, EVs/sEVs should no longer be considered as mere biological messengers or cell-free substitutes for stem cells, but be developed as therapeutic products [24,350,388].

## 10. Clinical Translation and Outlook

If the preceding section defines what still impedes clinical translation, the present section addresses where the field appears to be heading. At present, the evidence base is still mostly preclinical. Systematic reviews and meta-analyses support the idea that EVs can improve cardiac function and reduce injury in animal and in vitro myocardial injury models, but these findings should not be treated as proof of clinical efficacy [336,402]. More recent reviews also suggest that cardiac EV therapy is moving from a simple paracrine explanation toward a more product-oriented field, where the vesicle source, cargo, engineering method, and route of delivery all become part of the therapeutic design (see Figure 2) [348,403].

At the same time, the clinical horizon should not be overstated. True cardiac EV therapeutics remain largely preclinical, but the field has moved beyond purely exploratory biology. Reviews now report early-phase MSC-derived EV/sEV clinical activity in adjacent settings. GMP-oriented production of human CPC-derived EV/sEV products has been demonstrated, which is an important step because it shows that at least some cardiac EV products can be prepared with clinical manufacturing in mind [120]. Reviews of MSC-derived EVs/sEVs also describe early clinical trial activity in other disease areas [404]. Similarly, first-in-human cardiovascular secretome use in HF suggests that related cell-free biologics are beginning to enter early clinical exploration [405]. Patient-derived donor cell EV/sEV studies and clinically oriented biomaterial delivery syntheses further support the shift from proof-of-principle biology toward more translationally configured products and delivery paradigms by linking reparative EV biology to product design, manufacturing, and route of administration [353,406]. In parallel, recent patent activity around cardiac EV products, engineered vesicles, and EV-based delivery devices suggests that the field is also beginning to organize around protectable product formats, although patents should be interpreted as development signals rather than efficacy evidence [407,408,409,410,411,412,413,414,415]. Thus, the outlook for cardiac EV therapy is increasingly shaped by product development considerations.

### 10.1. Current Clinical Evidence and Trial Landscape

Although EV therapeutics have begun to enter human testing (see Figure 2) [416], clinical translation in CVD remains early [405]. Published therapeutic evidence is still concentrated mainly in non-cardiac indications, including coronavirus disease 2019 (COVID-19)-associated respiratory failure or acute respiratory distress syndrome [417,418,419], wound healing [420], dermatologic applications, including a topical MSC sEV ointment developed for psoriasis and evaluated in healthy volunteers [421], neurologic disease [422,423], and oncology [424,425]. These early studies generally support acceptable short-term feasibility and tolerability [417,418,419,420,421,422,423,424,425], but efficacy data remain preliminary and indication-specific, and not yet generalizable to cardiac repair. In CVD, the clinical landscape is more limited. The phase I clinical trial, Treatment of Non-ischemic Dilated Cardiomyopathies by Intravenous Infusions of the Extracellular Vesicle-Enriched Secretome of Cardiovascular Progenitor Cells (SECRET-HF) is evaluating repeated intravenous administration of an EV-enriched secretome derived from iPSC-derived cardiovascular progenitor cells in patients with non-ischemic dilated cardiomyopathy [405], and a separate coronary artery bypass grafting-related study is registered for MSC-derived sEVs combined with autologous mitochondria [426]. These studies are important development signals, but they should be distinguished from completed randomized efficacy trials of purified sEV therapeutics.

At the time of writing, no published randomized clinical evidence establishes therapeutic efficacy of EV or sEV products for MI or HF. Current cardiovascular EV studies include biomarker-focused work, including studies of EV RNA and miRNA cargo [427,428]. In adjacent vascular or neurovascular systems, a phase I Moyamoya disease study of iPSC-derived sEVs is registered in China [429], and the Stem Cell-derived Extracellular Vesicle Therapy in Acute Ischemic Stroke (STEVIA) study is listed as a South Korean acute ischemic stroke EV trial [430], but these studies do not yet provide published evidence for cardiac efficacy. Although the field has generated compelling mechanistic and animal data, translation has lagged because of manufacturing, characterization, dosing, potency, biodistribution, and regulatory challenges [431,432].

### 10.2. Patent Landscape and Product-Development Signals

The translational movement is visible in the patent landscape, although patent filings should be interpreted differently from efficacy studies. They do not prove clinical benefit, but they do show which product concepts are being protected and moved toward development. Several cardiac-focused patent families are centered on CPC-derived or cardiosphere-derived cell EVs, including claims for heart failure with preserved ejection fraction (HFpEF), ventricular tachyarrhythmias, and broader tissue regeneration applications [407,408,409]. Other filings move closer to defined cardiac indications, such as cardiac stromal cell-derived sEVs for ischemic heart repair and human adipose MSC-derived sEVs with high IL-10 expression for MI [410,412]. These patent families are consistent with the general trend that EV therapy is being framed less as a generic secretome effect and more as an indication-specific biologic product.

Patent literature also mirrors the shift toward engineering and delivery-focused platforms. Recent filings describe engineered EV compositions for targeted treatment of injured myocardium, including targeting and antisense oligonucleotide concepts, while sEV-eluting stent patents extend EV therapy into a device-based cardiovascular delivery format [411,413]. A broader cardiovascular vesicle patent family and a more recent secretome-containing composition patent further suggest that EV-containing products are being developed across several overlapping categories: purified vesicles, EV-enriched secretomes, engineered vesicles, and device-associated vesicle systems [414,415]. These filings show the practical direction of the field and that the translational path is not a single unmodified EV product, but a set of defined product formats matched to disease, route, cargo, and delivery setting.

### 10.3. Engineered EV Products

One major way forward is the emergence of deliberately engineered EV products (see Figure 2) [433,434]. In this context, the central issue is no longer whether EVs or sEVs can have therapeutic effects, but rather which product architecture best suits the biological problem. The reviews on targeted EV delivery in CVD now highlight that clinically useful EVs may require deliberate engineering, particularly if systemic administration is the goal and spontaneous myocardial accumulation is otherwise limited [349,435].

An emerging group of strategies is aimed at improving tissue targeting and retention through membrane- or surface-level engineering. These include platelet membrane-engineered EVs that use peripheral monocytes for cardiac targeting, ischemic myocardium-targeting peptides, surface-engineered cardiosphere-derived cell EVs, monocyte-mimetic EVs, and EVs engineered to express a cardiomyocyte-binding peptide [66,80,436,437]. Together, these studies reflect an important shift in translational logic. Rather than assuming that systemically administered vesicles will reach the heart in therapeutically meaningful amounts, they attempt to shape biodistribution beforehand, thereby turning delivery into a design variable [349,435].

A second and partly complementary group of strategies focuses more on cargo. In these studies, EVs are being developed as a programmable carrier whose therapeutic identity is defined by their intended payload and state of the producer cell. Examples include engineered sEV-mediated NOX4 siRNA delivery for cardiac hypertrophy, fibroblast activation protein aptamer-functionalized EV delivery of TGFβ1 siRNA to injured cardiac tissue, engineered EVs for viral myocarditis, targeted miR-590-3p-loaded sEV treatment after acute MI, ultrasound-targeted microbubble destruction-assisted delivery of miR-21-loaded EV-enriched preparations in chemotherapy-associated cardiotoxicity, and ultrasound-triggered delivery of stromal cell-derived factor 1-alpha (SDF-1α)-enriched ADSC sEVs after MI [72,81,381,438,439,440]. One related study used liposomal delivery of an inhibitor against miR-185-5p in dilated cardiomyopathy [71]. Other platforms have gone further by modifying the producer cell or fabrication method, including source cell activation, customizable loading, or fabrication strategies, including silicate-activated endothelial progenitor cell EVs, thin film hydration loading of cardiac sEV-derived vehicles, engineered nanovesicles, miR-222-engineered EVs in injectable hydrogels, and platelet-bioengineered human iPSC-sEVs for fibrotic sinoatrial node disease [441,442,443,444,445]. Together, these studies suggest that engineered EV and EV-inspired vesicle therapeutics are becoming less generic and more intentionally designed, with cargo composition, membrane features, and target tissue selected in advance rather than inferred retrospectively.

### 10.4. Biomaterial-Assisted Delivery and Route Selection

Biomaterial-assisted delivery may provide a parallel and highly pragmatic route toward clinical utility (see Figure 2), addressing one of the most practical limitations of EV therapy: insufficient product residence at the site of injury. This is especially relevant in MI and I/R injury, where the therapeutic target is spatially defined. Therefore, injectable alginate-collagen systems, peptide hydrogels, shear-thinning depots, conductive or pH-responsive matrices, black phosphorus-reinforced platforms, and pericardial hydrogel delivery strategies have been developed to improve retention, local concentration, and temporal release of EV therapeutics [22,204,352,353,373,374,442,446,447,448,449,450,451,452,453]. These systems can do more than just prolong the duration of exposure. Depending on their formulation, they may also provide electrical conductivity, mechanical support, environmental responsiveness, or minimally invasive delivery options, all of which can influence the way EV cargo is presented to the injured myocardium [373,446,452,453].

Patch- and scaffold-based approaches extend this logic by linking EVs to structural biomaterials that actively shape the post-infarct microenvironment and provide a more durable reparative niche [454]. Reviews on EV-biomaterial systems increasingly argue that these material platforms should be considered as part of the therapeutic product [22,352,447]. Meta-analytic synthesis of combination therapy with hydrogel-based products also suggests promising activity of hydrogel-based combination approaches in preclinical MI models [353]. Therefore, in cardiac indications in which tissue preservation is a critical variable, local biomaterial-supported delivery may be a promising translocation route.

These developments also influence how route of administration is being conceptualized. Local cardiac delivery is attractive because it improves retention and may reduce the dose needed for meaningful myocardial exposure, especially in MI, I/R injury, and anatomically localized fibrotic lesions [22,66,72,204,352,353,373,374,442,445,446,447,448,449,450,451,452,453,454]. At the same time, systemic delivery continues to be appealing for repeated dosing, broader cardiovascular syndromes, and indications in which extracardiac immune or vascular compartments form part of the therapeutic target. The growing literature on targeted and systemically administered EVs suggests that this route is increasingly being considered not only for convenience, but also as a serious translational option when biodistribution can be shaped rationally [18,66,72,80,349,381,435,436,437,438,439,440]. Therefore, localized and systemic EV therapy should probably be developed as different product types, with distinct engineering requirements, pharmacologic assumptions, and clinical use cases.

### 10.5. Personalized EV Therapeutics

A second path is the possibility that the next phase of translation may not just be engineered but also personalized (see Figure 2). Here, the term personalized should not be understood narrowly as an autologous product for every patient. Rather, it may include several related concepts: products selected according to the disease context, producer cell state, the injury phase, or the predicted responsiveness in patient-specific model systems. Systems biology and computational approaches may help integrate producer cell features, cargo profiles, recipient cell uptake, and functional responses when defining these more selective EV strategies [455]. This idea is supported by evidence that EV biology in cardiac disease is highly context-dependent. Mitochondrial transfer by EVs may expand the therapeutic scope beyond conventional miRNA signaling and points to metabolic rescue as a potentially relevant translational function in itself [310]. Similarly, mitochondria-rich EVs were evaluated ex vivo in patient-specific cardiomyocytes generated for the StEm cell iNjECtion in cAncer survivors (SENECA) trial, demonstrating how individualized in vitro systems can help to define EV responsiveness in clinically relevant settings [318].

Other studies also support the same idea from a donor- and disease-state perspective. CPC-derived EVs/sEVs from pediatric patients have been reported to carry reparative miRNA signatures, while EVs/sEVs derived from heart failure patients can exhibit impaired reparative activity associated with dysregulated miR-21-5p [406,456]. These observations suggest that donor state and disease state may represent more than sources of variability and could become part of future patient stratification strategies. This could lead to a more selective use of EV products: not one generic EV therapy for all cardiac injuries, but products chosen according to the biology of the injury and the therapeutic task.

### 10.6. A Realistic Near-Term Clinical Outlook

Overall, the outlook is becoming more concrete, but the field should remain disciplined. Cardiac EV therapeutics are not yet approved medicines, and the manufacturing, potency, biodistribution, safety, and regulatory challenges outlined in Section 9 remain substantial. Still, the field is no longer confined to proof-of-principle biology. It increasingly includes GMP-oriented manufacturing, updated clinical trial overviews, first-in-human cardiovascular experience with related cell-free biologics, engineered vesicles designed for tissue targeting or systemic delivery, biomaterial-enabled retention strategies, and more personalized concepts of cargo selection and patient matching [22,120,310,318,348,349,352,353,403,404,405,406,435,446,447,456]. At a broader level, regenerative medicine and bioengineering reviews suggest that EVs are being integrated into a wider therapeutic ecosystem that includes tissue engineering, advanced biomaterials, and next-generation cell-free regenerative platforms [457,458,459,460,461]. MSC EV literature further supports this general cell-free direction, although much of that evidence remains non-cardiac specific [462]. In addition, Wang et al. [463] showed that EVs from young MSCs, but not unmodified aged MSCs, rejuvenated senescent endothelial progenitor cells in vitro. miR-126-transduced aged MSCs subsequently generated EVs with rejuvenating activity in vitro and angiogenic activity after ischemic injury comparable to that of young MSC EVs. This finding identifies donor age and producer cell engineering as product-defining variables rather than supporting MSC EVs as a uniform therapeutic class.

The cardiac EV products most likely to succeed clinically will probably be those that integrate four features in a coherent way: a defined producer cell source, mechanism-linked cargo, route-appropriate targeting, and a delivery format matched to the specific disease. The next step is to define those conditions more rigorously. If that integration can be achieved, personalized EV-based cardiac medicine may become realistic, not because every patient receives the same EV product, but because the vesicle product can be matched to the biology of the injury and to the therapeutic goal.

## 11. Conclusions

Small extracellular vesicles are a promising but still heterogeneous class of cell-free therapeutic preparations for cardiac injury. The current evidence is strongest for cardioprotection and cardiac repair, including limitation of cell death, support of vascular recovery, modulation of post-injury inflammation, and restraint of adverse remodeling. In contrast, regeneration should remain a stringent evidentiary claim that requires demonstration of durable newly formed myocardium and functional integration rather than improved ventricular function or reduced scar burden alone. Accordingly, improvements in ventricular function, remodeling, or symptoms are described as functional recovery and should not, without additional evidence, be interpreted as myocardial regeneration.

Collectively, preclinical studies support continued development of EV-based cardiac therapeutics, but no EV/sEV product has established clinical effectiveness for myocardial infarction or heart failure. Future progress will depend on defining EV products by their producer cell source, manufacturing process, characterization profile, a justified and standardized dose metric, mechanism-linked potency and release criteria, route of delivery, biodistribution and safety, and intended clinical indication. At present, no field-wide standardized dose metric exists, and particle counts, protein mass, and administered volume should not be treated as interchangeable. Regulatory RNAs, proteins, lipids, and organelle-associated material may contribute to benefit, but their individual roles should be distinguished from cargo association alone and supported by appropriately rigorous causal evidence. Aligning biological mechanism with reproducible product definition and clinically realistic delivery will be essential for translating the most credible preclinical observations into cardiac therapeutics.

## Figures and Tables

**Table 1:** A critical comparison of EV/sEV source classes relevant to cardiac therapy. Abbreviations: MSC, mesenchymal stem cell; iPSC, induced pluripotent stem cell; iPSC-CM; induced pluripotent stem cell-derived cardiomyocyte; iPSC-EC, induced pluripotent stem cell-derived endothelial cell; CPC, cardiac progenitor cell; 3D, 3-dimensional.

Source	Principal Rationale	Critical Limitations	Translational Interpretation
MSC	Deepest preclinical base; practical expansion	Donor tissue, age, passage, senescence, priming; heterogeneous products	High, but conditional on scalable culture and product definition
iPSC-derived	Renewable or clonal producer potential; engineering flexibility	Differentiation state; residual pluripotency-associated material; lineage mixing	Potentially high; requires stringent identity and safety controls
iPSC-CM/iPSC-EC	Cardiac- or vascular-lineage signals	Complex differentiation; lower yields; sparse matched comparisons	Promising but early
CPC/cardiosphere	Cardiac-aligned rationale; early manufacturing experience	Variable producer definitions; smaller and less standardized evidence base	Moderate/uncertain
Immune/plasma/tissue	Captures systemic or disease-relevant signaling	Strong state dependence; mixed origins; potentially harmful cargo; purification burden	Useful for mechanism/biomarkers; therapeutic sourcing difficult
Cardiac organoid/3D	Multicellular context; potential tissue-like cargo; potency model value	Very early evidence; scale, matrix, composition drift, batch definition	Emerging platform, not established source

**Table 2:** Representative preclinical studies of administered EV/sEV products in cardiac injury. This table summarizes representative preclinical studies across MI, I/R, chemotherapy-associated injury, hypertrophic remodeling, myocarditis, diabetic injury, and organoid systems, highlighting that efficacy depends not only on vesicle source but also on model context, delivery route, timing, and formulation design. The listed studies include exogenous therapeutic EV administration, preconditioning/prophylactic designs, endogenous or treatment-induced EV signaling studies, and biomaterial-combined platforms. These intervention contexts should not be interpreted as equivalent when comparing apparent efficacy. Abbreviations: AMI, acute myocardial infarction; LAD, left anterior descending; MSC, mesenchymal stem cells; EV, extracellular vesicle; sEVs, small extracellular vesicles; TEM, transmission electron microscopy; NTA, nanoparticle tracking analysis; EF, ejection fraction; FS, fractional shortening; I/R, ischemia/reperfusion; NanoFCM, Nano flow cytometry; NR, not reported; PBS, phosphate-buffered saline; HUVEC, human umbilical vein endothelial cells; BCA, bicinchoninic acid; PDCD4, programmed cell death protein 4; PTEN, phosphatase and tensin homolog; FasL, Fas ligand; PELI1, pellino E3 ubiquitin protein ligase 1; ALIX, ALG-2-interacting protein X; TSG101, tumor susceptibility gene 101 protein; MI, myocardial infarction; TXL, Tongxinluo; eNOS, endothelial nitric oxide synthase; CM, cardiomyocyte; CK-MB, creatine kinase MB; ISO, isoproterenol; DLS, dynamic light scattering; FESEM, field emission scanning electron microscopy; ROS, reactive oxygen species; NF-κB, nuclear factor kappa-light-chain-enhancer of activated B cells; TGF-β1, transforming growth factor-β1; SMAD3, suppressor of mothers against decapentaplegic family member 3; TAC, transverse aortic constriction; HFrEF, heart failure with reduced ejection fraction; IL-4, interleukin 4; SV, stroke volume; LVESV, left ventricular end-systolic volume; IFN, interferon; LVEDD, left ventricular end-diastolic diameter; LVFS, left ventricular systolic function; LVID, left ventricular internal dimension; HF, heart failure; BMSC, bone marrow stem cell; IMTP, ischemic-myocardium-targeting-peptide; BNP, B-type natriuretic peptide; cTnI, cardiac troponin I; Ca^2+^, calcium; BMMSC, bone marrow-derived mesenchymal cells; MIF, macrophage migration inhibitory factor; TNF-α, tumor necrosis factor-α; LPS, lipopolysaccharide; HDL, high density lipoprotein; SCFAs, short chain fatty acids; SEM, scanning electron microscopy; LV, left ventricle; α-SMA+, alpha-smooth muscle actin positive; FAC, fractional area change; iPSC, induced pluripotent stem cell; Lamp2b, lysosome-associated membrane protein 2b; ESC, embryonic stem cell; hTERT, human telomerase reverse transcriptase; DiI, 1,1′-dioctadecyl-3,3,3′-tetramethylindolecarbocyanine perchlorate; TXNIP, thioredoxin-interacting protein; CASP1, caspase-1; NLRP3, NOD-like receptor protein 3; AAR, area at risk; SEC, size exclusion column; ECG, electrocardiogram; MVO, microvascular obstruction; CMR, cardiovascular magnetic resonance; NET, neuroendocrine tumor; RIHD, radiation-induced heart disease; Dox, doxorubicin; Trz, trastuzumab; ANP, atrial natriuretic peptide; Ang II, angiotensin II; qRT-PCR, quantitative reverse transcriptase polymerase chain reaction; β-MHC, beta-myosin heavy chain; UCMSC, umbilical cord mesenchymal stem cell; siRNA, small interfering RNA; VCAM-1+, vascular cell adhesion molecule 1 positive; IL-1β, interleukin-1beta; IFN-γ, interferon-gamma; PAK2, p21 activated kinase 2; ERK1/2; extracellular signal-regulated kinase 1/2; WT, wild type; i.p., intraperitoneal; i.v., intravenous; LSM, laser scanning microscopy; EMMPRIN, extracellular matrix metalloproteinase inducer; D_2_O, deuterium oxide; kDa, kilodalton; PEG, polyethylene glycol; NaCl, sodium chloride; BMP2, bone morphogenetic protein 2; AFM, atomic force microscopy; Hsp90, heat shock protein 90; Hsp70, heat shock protein 70; EEA1, early endosome antigen 1; GPVI, glycoprotein VI; MIRI, myocardial ischemia/reperfusion injury; HEPES, 4-(2-hydroxyethyl)-1-piperazineethanesulfonic acid; CYP, cyclophosphamide; HW/BW, heart weight to body weight ratio; STZ, streptozotocin; cTnT, cardiac troponin T; CVB3, Coxsackievirus B3; dLN, draining lymph node; DC, dendritic cells; dSTORM, direct stochastic optical reconstruction microscopy; ELISA, enzyme-linked immunosorbent assay; PEV, platelet-derived extracellular vesicles; pmPEV, premenopausal extracellular vesicles.

Reference	Model/Species	EV Source/Platform	Isolation/Characterization	Dose and Normalization	Route/Timing/Follow-Up	Major Outcome
[30]	AMI, permanent LAD artery, Rat	Normoxic and 48 h hypoxia-preconditioned human umbilical cord MSC sEVs; hypoxic sEVs depleted of miR-214	Total Exosome Isolation reagent after 2000× *g* clarification and 0.22 µm filtration; TEM, NTA, and immunoblotting for CD63/TSG101	40 µg in 40 µL PBS	Intramyocardial injection at two ischemic border sites immediately after LAD artery ligation; echocardiography on days 1, 7, 14, and 28; terminal analysis day 28	Hypoxia preconditioned sEVs outperformed normoxic sEVs with improved EF/FS, ↓ fibrosis, ↑ CD31-positive capillary density
[73]	I/R, Rat & pig	M2 macrophage-derived sEVs	Differential ultracentrifugation; TEM and NanoFCM; particles < 150 nm	150 µg total protein in 100 µL (rat); 1.0 mg in 500 µL (pig)	Five-site intramyocardial injection 30 min after reperfusion; follow-up NR	Improved cardiac function; ↓ infarct/scar markers, CCR2^+^ macrophages/monocytes; ↑ M2 polarization/neovascularization
[39]	AMI, permanent LAD artery, Mouse	HUVEC-derived sEVs	Differential ultracentrifugation; TEM, NTA, and immunoblot (TSG101/CD63/CD81; calnexin-negative)	50 µg total protein (25 µL at 2 µg/µL)	Localized intramyocardial injection immediately after LAD artery ligation; follow-up at days 7 and 28	Improved cardiac function; ↓ infarct size, fibrosis, apoptosis
[41]	I/R, Mouse	Bone marrow MSC-derived sEVs	Differential ultracentrifugation (2000× *g* for 20 min, 10,000× *g* for 30 min, and 100,000× *g* for 70 min); BCA protein assay; TSG101/CD9 and vesicle size reported; PKH26/flow-imaging uptake analyses	0.5 µg/µL at 1 µL/g body weight; 12.5 µg protein (~5.62 × 10^5^ vesicles) for a 25 g mouse	Pericardial sac injection 24 h pre-I/R; infarct size at 24 h after I/R and target/caspase analyses at 6 h	WT sEVs: ↓ infarct size and PDCD4/PTEN/FasL/PELI1
[61]	AMI, permanent LAD artery, Mouse	M2 macrophage-derived sEVs	2500× *g* for 30 min, 8500× *g* for 30 min, and 120,000× *g* for 70 min twice, followed by PBS wash/third 70 min spin and 0.22 µm filtration; BCA, NTA, TEM, and ALIX/TSG101/CD63 immunoblot with GM130-negative control	20 µg total in 30 µL; single administration	Three-site intramyocardial injection at onset of MI; echocardiography and histology on day 28	Improved cardiac function; ↓ infarct size; ↑ angiogenesis
[62]	MI, permanent LAD artery, Mouse	Mouse M1-like macrophage-derived sEVs	Differential ultracentrifugation (300× *g*, 2000× *g*, 10,000× *g*, and 120,000× *g*) with 0.22 µm filtration and PBS wash; TEM, NTA, and immunoblotting for CD63/CD81; calnexin negative control	In vivo dose NR in the supplied main article; dosing information may have been confined to the unavailable supplement	Intramyocardial administration after permanent LAD artery ligation; administration time NR; angiogenesis assessed on days 14 and 28; cardiac function, infarction, and survival assessed on day 28	Worsened survival and EF/FS; ↑ infarct size; ↓ angiogenesis markers
[59]	I/R, Rat	Human cardiomyocyte-derived sEVs, especially TXL-pretreated CM sEVs	300× *g* for 10 min, 2000× *g* for 20 min, 13,500× *g* for 30 min, and 120,000× *g* for 70 min twice; TEM, NTA, and CD81/CD63 immunoblot with calnexin negative control	No EV product was administered	TXL 0.4 g/kg by gavage 1 h before I/R; GW4869 1 mg/kg i.p. every 24 h for 3 days before I/R; 45 min ischemia/3 h reperfusion	Improved microcirculation; ↓ infarct size and CK-MB; ↑ eNOS activity
[67]	ISO-induced cardiac fibrosis, Rat	Rat adipose-derived MSC sEVs, untreated or produced after 80 µM rosmarinic-acid priming for 72 h	Commercial Exocib precipitation kit after double 0.22 µm filtration; exosomal protein quantified by Bradford/BCA-type assay; CD9/CD63 flow cytometry, DLS, and FESEM	100 µg/mL sEV preparation per i.v. injection; injected volume and total protein dose per rat NR. The same concentration was used in vitro	Tail vein injection beginning 24 h after the second ISO dose on protocol days 1, 7, 14, and 21; ECG on day 2; final echocardiography and tissue collection on day 28	Improved cardiac function; ↓ CK-MB/troponin I, ROS, ST elevation, NF-κB/TGF-β1/SMAD3/collagen; ↑ TAC
[79]	Diet-induced occlusive coronary atherosclerosis with MI/HFrEF, Mouse	Human THP-1 IL-4-polarized M2 macrophage sEVs	400× *g* for 10 min, 2000× *g* for 20 min, 0.2 µm filtration, and cushioned density gradient ultracentrifugation (60% OptiPrep, 100,000× *g* for 3 h, then 5–20% gradient at 100,000× *g* overnight); fraction-7 dialysis; NTA and TEM	1 × 10^10^ particles per i.p. injection; three injections weekly for 4.5 weeks; exact cumulative number not explicitly stated	Paigen diet for 4.5 weeks to induce MI/HF, followed by pIpC lipid lowering and i.p. sEV injection 3 times weekly for 4.5 weeks; terminal follow-up after 4.5 weeks of treatment	Reversed HFrEF decline; ↑ EF/FS/SV; ↓ LVESV, remodeling, inflammation, type I IFN signaling
[316]	I/R, Mouse	Cardiac-derived sEVs and ATP5a1-overexpressing mouse adipose-MSC EVs	Heart EVs: collagenase digestion; 300× *g* for 5 min, 2000× *g* for 10 min, 10,000× *g* for 10 min, and 120,000× *g* for 2 h twice. Adipose-MSC EVs: 2000× *g* for 30 min, 10,000× *g* for 30 min, and 120,000× *g* for 70 min twice; NTA/LSM, TEM, and TSG101/ALIX/CD9 immunoblot with calnexin negative control	2.0 × 10^9^ particles/µL in 50 µL (1.0 × 10^11^ particles) per mouse; single administration	Two site intramyocardial injection immediately after reperfusion; echocardiography on days 3 and 28; terminal follow-up day 28	Cardiac-derived sEV: ↑ EF/FS; ↓ LV volumes, infarct, inflammation/remodeling, ferroptosis, mitochondrial damage
[117]	MI, permanent LAD artery, Mouse	Human CPC-derived sEVs	Differential centrifugation ending at 110,000× *g*; sucrose gradient separation (190,000× *g* for 16 h and 110,000× *g* wash); BCA, qNano, TEM, and immunoblotting for flotillin-1/CD9/CD63/CD81/ALIX/TSG101/EMMPRIN with calnexin negative control	8 µg in 10 µL total (0.8 µg/µL), delivered as two 5 µL injections	Intramyocardial border zone injection 15 min after LAD artery ligation; terminal follow-up at 2 days	↓ Infarct size; ↑ cardiovascular cell proliferation
[354]	I/R, Rat	Native, blank vector, and IMTP-engineered human umbilical cord MSC sEVs	0.22 µm filtration, 2000× *g* for 30 min, 10,000× *g* for 45 min, refiltration through 0.45 µm, and 100,000× *g* for 70 min; TEM, NanoFCM/NTA, and CD63/CD9 immunoblot	400 µg in 200 µL (~1.5 × 10^10^ particles) per i.v. dose; five administrations; cumulative 2.0 mg and ~7.5 × 10^10^ particles	Tail vein immediately after reperfusion and on days 3, 5, 14, and 28; assessments at 24 h, 4 weeks, and 3 months; terminal follow-up at 3 months	All sEVs reduced injury, IMTP-engineered sEV showed more ↓ apoptosis, oxidative stress, inflammation, fibrosis; improved EF/FS/LVEDD; ↑ angiogenesis
[355]	AMI, permanent LAD artery, Rat	Human umbilical cord MSC-derived sEVs	300× *g* for 20 min, 2000× *g* for 20 min, 10,000× *g* for 30 min, 100 kDa concentration, 30% sucrose/D_2_O cushion at 100,000× *g* for 2 h, 100 kDa PBS washes, and 0.22 µm filtration; BCA, TEM, NTA, and CD9/CD63 immunoblot	400 µg protein in 200 µL PBS; single infusion	Tail vein infusion immediately after LAD artery ligation; assessments at 2 days, 1 week, and 4 weeks; terminal follow-up at 4 weeks	↑ LVEF/LVFS; ↓ LVIDs, fibrosis, apoptosis
[356]	AMI, permanent LAD artery, Mouse	Cardiac MSC-derived sEVs	1000 rpm for 10 min, 0.22 µm filtration, overnight PEG-4000/NaCl precipitation, and 3000 rpm for 30 min; NTA, immunogold TEM/CD63, and immunoblotting for TSG101/CD81/CD63	50 µg in 30 µL PBS; single administration	Intramyocardial border zone injection immediately after LAD artery ligation; echocardiography at baseline, day 1, and day 30; terminal follow-up at 1 month	Preserved EF/function; ↑ capillary density and viable myocardium; thicker scar
[357]	AMI-induced HF, Rat	Rat BMSC-derived EVs including unmodified, BMP2-overexpressing, and BMP2-knockdown preparations	Total Exosome Isolation reagent after hypoxic conditioning, 2000× *g* clarification, 0.2 µm filtration and 100 kDa concentration; TEM, qNano/tunable resistive-pulse sensing, and immunoblotting for CD63/CD81/TSG101; calnexin negative control	Unmodified EV group: 50 µg in 30 µL PBS. Engineered EV groups received 50 µL; protein mass NR	Intramyocardial injection at five peri-infarct sites, reported as 30 min after LAD artery ligation; final assessment at 6 weeks. The stated 30 min timing conflicts with the paper’s description of selecting HF animals by LVEF later in the model	Unmodified BMSC EVs: ↑ EF/FS/hemodynamics; ↓ BNP/cTnI, fibrosis, inflammation, apoptosis; ↑ angiogenesis. BMP2-overexpressing EVs produced greater benefit and BMP2 knockdown attenuated protection
[116]	MI, permanent LAD artery, Mouse	Human iPSC endothelial cell-derived sEVs	Low speed clarification, 0.22 µm filtration, and ExoQuick precipitation; NTA, TEM, and immunoblot (ALIX/TSG101/CD63/CD9)	20 µg total protein in 20 µL PBS	Three site intramyocardial injection immediately after LAD artery ligation; follow-up at 3, 14, and 28 days	↑ LVEF/LVFS/hemodynamics; ↓ infarct/scar, apoptosis, hypertrophy; preserved Ca^2+^ handling; no excess arrhythmias
[358]	AMI, permanent LAD artery, Rat	Human BMMSC sEVs including MIF-overexpressing BMMSC sEVs and control BMMSC sEVs	Ribo Exosome Isolation Reagent precipitation after culture with exosome depleted serum; BCA protein assay, NTA, and CD63/CD81 assessment by flow cytometry/immunoblotting	30 µg exosomal protein in 30 µL PBS	Intramyocardial injection at four infarct border sites immediately after permanent LAD artery ligation; follow-up at 4 weeks	MIF-overexpressing BMMSC sEVs more effective in ↑ LVEF/LVFS; ↓ scar/fibrosis, ROS, mitochondrial fragmentation, apoptosis
[359]	MI, permanent LAD artery, Mouse	TNF-α-treated bone marrow MSC-derived sEVs	500× *g* for 10 min, 12,000× *g* for 20 min, and 100,000× *g* for 2 h; NTA and immunoblotting for CD63/CD9/ALIX	5 µg in 25 µL PBS; single administration	Five-site intramyocardial injection within 30 min of LAD artery ligation; terminal follow-up at day 28	↓ LDH/CK-MB/troponin I, LVIDd/s, infarct; ↑ LVEF/LVFS, LV wall; M2 > M1 polarization
[360]	MI, permanent LAD artery, Mouse	Human placental MSC-derived sEVs	Differential centrifugation/ultracentrifugation with PBS washes (up to 100,000× *g*); TEM and NTA	200 µL per i.v. injection; protein/particle dose NR; three administrations	Tail vein injection on post-MI days 7, 9, and 11; terminal follow-up at 4 weeks after MI	↓ fibrosis/remodeling, injury/lipid/inflammatory markers, LPS; ↑ HDL/SCFAs; anti-inflammatory microbiota shift
[361]	MI, permanent LAD artery, Rat	Human endometrial MSC-derived sEVs	300× *g* for 10 min, 2000× *g* for 10 min, 10,000× *g* for 30 min, and Exocib C kit; Bradford protein assay, DLS, SEM, and immunoblotting for CD9/CD63/CD81	300 µL at 1 µg/µL (300 µg) per rat, free or fibrin encapsulated	Intramyocardial injection at three peri-infarct sites immediately after MI; echocardiography at baseline, 24 h, and 30 days; terminal day 30	↓ fibrosis/infarct/remodeling/inflammation; ↑ angiogenesis, LV function, cardiomyocyte retention
[362]	MI, permanent LAD artery, Rat	Human umbilical cord MSC sEVs carrying circRNA 0001273	2000× *g* for 20 min followed by overnight commercial precipitation and 1 h centrifugation; BCA; flow-cytometric CD63 and immunoblotting for CD9/CD63/CD81 with calnexin; DiI uptake	100 µL exosome preparation; concentration reported as 5 µg/L, an internally implausible unit, so total protein dose cannot be determined reliably	Intramyocardial administration after MI induction; echocardiography on days 1, 3, 7, 14, and 28; final day 28	↑ LVEF/LVFS; improved myocardial histology
[363]	MI, permanent LAD artery, Rat	Empagliflozin pretreated MSC-derived sEVs	1500× *g* clarification; Ribo Exosome Isolation Reagent precipitation (12 h at 4 °C; 2000× *g*); NTA, TEM, and immunoblotting for CD63/CD81/TSG101	sEVs produced by 1 × 10^6^ MSCs in 50 µL PBS per rat; mass/particle dose NR	Three site intramyocardial injection after LAD artery ligation; echocardiography on days 23 and 36; final histology on day 36	↑ EF/FS, angiogenesis, BCL-2; ↓ infarct, fibrosis, inflammation, apoptosis
[113]	Ischemic cardiomyopathy after LAD artery, Rat	Human iPSC-derived cardiomyocyte EVs	Conditioned medium filtration, modified polyethylene glycol concentration, and ultracentrifugation; NTA; CD63/syntenin immunoblot and Exo-Check marker array	1.0 × 10^10^ particles in 100 µL; 20 µL at each of 5 peri-infarct sites	Intramyocardial injection 2 weeks after LAD artery ligation; longitudinal assays through 4 weeks after treatment (6 weeks after MI)	↑ LVEF/LV dimensions/survival, angiogenesis, reparative genes; ↓ fibrosis; ↑ M2 polarization
[364]	MI, permanent LAD artery, Mouse	Pericardial adipose tissue-derived sEVs	Tissue explant conditioned medium; 1000× *g* for 5 min and 10,000× *g* for 10 min, 0.22 µm filtration, ultracentrifugation or ExoQuick; NTA, TEM, and immunoblotting for CD63/TSG101 with GM130 negative control	100 µg exosomal protein per i.v. injection; four injections; cumulative: 400 µg	Tail vein injection on post-MI days 1, 3, 5, and 7; echocardiography and terminal analysis on day 28	↑ survival and LVEF/LVFS; ↓ fibrosis, ferroptosis/lipid oxidation; improved iron homeostasis
[365]	AMI, permanent LAD artery, Mouse for efficacy; rat for retention and delivery feasibility; healthy pig for procedural feasibility and safety	MSC-derived sEVs in fibrin spray scaffold	MSC secretome concentrated with 3 kDa ultrafiltration; sEVs concentrated and washed with 100 kDa filters; NanoSight used for size/concentration and SEM for the fibrin-EV formulation	Mouse and rat sEV doses NR; pig received 5 mL EXOS formulation	Mouse: epicardial fibrin EXOS spray immediately after MI; echocardiography at 4 h and day 28. Rat: thoracoscopic spray/retention assessment at 24 h. Healthy pig: thoracoscope-guided intrapericardial spray; safety assessment on day 7	Mouse MI: ↑ LVFS/LVEF, LV wall, viable myocardium, angiomyogenesis markers; ↓ infarct/scar
[366]	AMI, permanent LAD artery, Rat	Human MSC-derived EVs, free or encapsulated in RADA4-SDKP self-assembling peptide hydrogel	Differential ultracentrifugation at 2500× *g*, 20,000× *g*, and 100,000× *g* with PBS washing; BCA assay, SEM, DLS, and immunoblotting for CD9/CD81/CD63/TSG101; calnexin negative control	80 µg MSC-EVs in 100 µL per rat, either free or hydrogel encapsulated	Intramyocardial injection at four infarct border sites during AMI surgery; echocardiography on days 1 and 28; terminal analysis at week 4	↑ LVEF/FS; ↓ LVIDs, scar/fibrosis, CD68^+^ macrophages; ↑ α-SMA^+^ vessels
[367]	I/R, Rat	CPC-derived sEVs	Serum-free conditioned medium; sequential ultracentrifugation; NTA and TEM	5 µg/kg in 100 µL PBS; single administration	Ultrasound guided intramyocardial injection into the ischemic zone 2 weeks after I/R; EF/FS at days 0, 7, 14, and 28 after injection; final histology on day 28 (6 weeks post-injury)	↑ EF/FS, angiogenesis, M2-like macrophages; ↓ fibrosis, degeneration, hypertrophy, TNF-α, ROS
[122]	I/R, Mouse	Human primary BM-MSC-, hTERT-MSC-, ESC-, ESC-CPC-, ESC-CM-, and ventricular cardiac fibroblast-derived sEVs	Serial ultracentrifugation for functional studies; SEC for yield analysis; TEM, NTA, and immunoblot (ALIX/TSG101/CD63/CD9/CD81; calnexin negative)	Normalized by total sEV protein; in vivo amount NR	Intramyocardial injection post-I/R; follow-up NR	ESC-sEV most effective in ↑ LVEF/FAC and vascular density; ↓ LV dilation/fibrosis; strongest angiogenic/anti-fibrotic profile
[110]	I/R, Mouse	Mouse iPSC-derived EVs	Serum-/feeder-free iPSC conditioned medium; sequential centrifugation including ultracentrifugation; NTA, AFM, high resolution flow cytometry, and immunoblotting for CD9/CD81; includes microvesicle and exosome fractions	NR	Intramyocardial injection 48 h after reperfusion; echocardiography 48 h after injection and day 35 after MI; terminal follow-up on day 35	Improved LV function/remodeling vs. vehicle/iPSCs; ↑ angiogenesis/viable myocardium; ↓ apoptosis; no teratomas
[368]	post-MI HF, permanent LAD artery, Rat	Endogenous serum sEVs	Serum/culture media serial centrifugation (2000× *g* and 10,000× *g*, followed by the reported 10,000× *g* for 3 h) with PBS wash; BCA protein assay; no morphology or EV marker panel reported	No EV product was administered	Five cycles of 5 min bilateral hindlimb ischemia/5 min reperfusion once daily for 4 weeks, beginning 4 weeks after MI; terminal follow-up at 8 weeks after MI	↑ LVEF/diastolic function; ↓ LV volumes, fibrosis, remodeling, oxidative stress
[66]	AMI, permanent LAD artery, Mouse	Mouse bone marrow MSC sEVs: control, empty vector, and Lamp2b–CSTSMLKAC IMTP-engineered sEVs	Total Exosome Isolation reagent after 500× *g* clarification; PBS resuspension; TEM, NTA, and immunoblotting for TSG101/CD63/CD9 and Lamp2b, with cellular protein contamination assessment	50 µg in 100 µL PBS (reported as 4 × 10^9^ particles) per mouse; single i.v. dose	Tail vein immediately after LAD artery ligation; biodistribution through 72 h; echocardiography: 0, 3, 14, and 28 days; histology: days 3 and 28; terminal day 28	IMTP-engineered sEV improved heart targeting, EF/FS/remodeling; ↓ inflammation, apoptosis, fibrosis; ↑ vasculogenesis
[18]	AMI, permanent LAD artery, Mouse	Human CDC-derived sEVs	0.2 µm filtration, 10 kDa ultrafiltration, qEV1 70 nm SEC, pooling fractions 1–5, and 10 kDa reconcentration; BCA, NTA, zeta potential, cryo-TEM, and immunoblotting for ALIX/CD81/Hsp90/TSG101 with calnexin/cytochrome-c/EEA1 controls	100 µg EV protein per mouse; intramyocardial: 20 µL across two 10 µL peri-infarct injections; i.v.: 100 µL tail vein injection; single dose	Intramyocardial or i.v. injection immediately after LAD artery ligation; biodistribution at 2 and 24 h; echocardiography: days 2 and 28; terminal day 28	CMP-EVs: ↑ cardiac retention and LVEF; ↓ infarct, apoptosis, fibrosis, macrophage infiltration
[369]	I/R, Mouse	Anoxia preconditioned adipose MSC sEVs	2000× *g* for 30 min, 100,000× *g* for 70 min, PBS resuspension, and repeat 100,000× *g* wash; NanoSight, TEM, and immunoblotting for TSG101/CD9 with calnexin negative control	10 µg/g body weight by tail vein; single administration	Tail vein injection before I/R surgery; exact pre-I/R interval NR; infarct/AAR and molecular/apoptosis readouts at 4–12 h, echocardiography immediately after surgery and days 3 and 7; terminal day 7	Int-EVs: ↓ infarct/apoptosis and TXNIP/CASP1/NLRP3 signaling; ↑ EF/FS
[370]	I/R, Mouse	Human plasma-derived EVs and platelet membrane-fused hEV	SEC followed by 100 kDa centrifugal concentration; platelet membrane fusion by repeated extrusion; TEM, NTA, zeta potential, and immunoblotting for ALIX/TSG101/CD63/ApoA1 and platelet markers CD42b/GPVI/CD47	200 µL at 5 × 10^10^ particles/mL, equivalent to 1 × 10^10^ particles per injection	Tail vein injection 10 min after reperfusion; repeat injection every 7 days for the long-term experiment; acute assessment at 24 h and final assessment at 3 weeks	Improved injured heart targeting and EF/FS; ↓ infarct, apoptosis, fibrosis/remodeling; ↑ CD31^+^ vessels
[371]	I/R, Mouse	Bone marrow MSC-derived sEVs	3000× *g* for 30 min, 10,000× *g* for 1 h, and 50,000× *g* for 2 h; PBS resuspension; TEM, NTA, and immunoblotting for Hsp70/CD63/TSG101	3 µg/g body weight in 100 µL PBS per mouse; single dose	Caudal vein injection immediately after reperfusion; acute assays: 6–72 h; infarct/MVO: day 3, CMR: day 7, echocardiography at 24 h, day 3, day 7, 1 month, fibrosis at 1 month; terminal at 1 month	↓ neutrophil infiltration/NET formation; attenuated I/R injury
[372]	I/R, Rat	Rat plasma-derived sEVs carrying miR-130a-3p	Commercial plasma EV extraction kit; TEM, NTA, and immunoblotting for ALIX/TSG101/CD9/CD63/CD81; calnexin negative control	25 µL at 2 µg/µL, equivalent to 50 µg per administration	Intramyocardial administration post-I/R, repeated every 3 days for 3 weeks; final assessment after the 3-week regimen	↑ LVEF/LVFS; ↓ infarct, necrosis/inflammation, fibrosis, excessive autophagy
[373]	I/R, Rat	Rat bone marrow MSC-derived sEVs plus metformin in injectable pH-responsive conductive hydrogel	Commercial exosome kit (specific kit/method NR); TEM, particle-size analysis, and immunoblotting for CD9/CD63/TSG101	50 µg MSC-sEV protein in 100 µL hydrogel; Met+Exo+Gel also contained 100 µg metformin; single injection	Intramyocardial border zone injection immediately after reperfusion/MIRI confirmation; apoptosis: day 3, exosome retention: day 14; final echocardiography/histology/arrhythmia/vascular outcomes: day 28	Met+Exo+Gel: ↓ infarct/fibrosis, apoptosis, arrhythmias; ↑ EF/FS, remodeling, conduction, reparative markers
[374]	I/R, Rat	Human umbilical cord MSC sEV-anchoring conductive hydrogel	0.45 µm filtration and differential ultracentrifugation at 300× *g*, 2000× *g*, 10,000× *g*, and 120,000× *g*; BCA assay, TEM, DLS/zeta potential, and immunoblotting for ALIX/CD63/CD9	150 µg exosomal protein in a total injection volume of 75 µL	Intramyocardial injection at three sites after 1.5 h LAD artery ischemia and reperfusion; retention assessed on days 3, 7, and 14; echocardiography on days 3, 7, 14, and 28; final analysis on day 28	↑ EF/FS and LV geometry/wall thickness; ↓ fibrosis/inflammation
[375]	Sequential Dox/Trz cardiotoxicity, Rat	sEVs from human cardiac-resident mesenchymal progenitor cells isolated from atrial appendages; preparations pooled from five to six donors	Differential ultracentrifugation after 3000× *g* and 10,000× *g* clarification and 0.2 µm filtration; NTA and flow cytometry for CD63/CD9/CD81/EpCAM	3 × 10^10^ particles in 0.1 mL per injection; three injections; cumulative dose 9 × 10^10^ particles	i.v. tail vein on days 5, 11, and 19; Dox on days 1–11; Trz on days 19–28; echocardiography at baseline, day 19, and day 37; final follow-up on day 37	Preserved LV function; ↓ LVESV, fibrosis, CD68^+^ infiltrates, iNOS, ROS
[376]	Sequential Dox/Trz cardiotoxicity, Rat	Rat adipose-derived MSC sEVs	2000× *g* for 20 min, 100,000× *g* for 1 h, serum-free M199/HEPES wash, and repeat 100,000× *g*; Bradford assay, TEM, and immunoblotting for CD63/CD81/CD83	3 × 10^10^ particles in 0.1 mL PBS per injection. Protective: three doses, cumulative 9 × 10^10^; curative: two doses, cumulative 6 × 10^10^	i.p. Dox: six injections over 2 weeks then 1-week interval, then Trz six injections over 2 weeks. Protective: one EV dose 1 week before induction plus two doses beginning 6 weeks after final Trz, 2 weeks apart; curative: two doses beginning 6 weeks after final Trz, 2 weeks apart; terminal 4 weeks after last EV dose	↓ injury markers, apoptosis/oxidative injury; ↑ LVEF/FS, histology/ultrastructure, pro-survival signaling
[377]	CYP-induced cardiotoxicity, Rat	Rat adipose-derived MSC sEVs	Exosome depleted serum; conditioned medium clarified twice at 500× *g* then 2000× *g* and 10,000× g, filtered at 0.22 µm, and ultracentrifuged twice at 110,000× *g*; BCA, TEM, DLS, and immunoblotting for TSG101/CD63/CD81 with calnexin negative control	3 × 10^10^ particles in 0.1 mL PBS per i.p. injection; two injections, cumulative 6 × 10^10^ particles	i.p. injection 1 week before CYP and again 1 week later on the CYP-injection day; terminal assessment 1 week after CYP	Reduced CYP cardiotoxicity
[378]	Dox-induced dilated cardiomyopathy, Mouse	Trophoblast stem cell-derived sEVs	300× *g* for 10 min, 2000× *g* for 20 min, 10,000× *g* for 30 min, and 100,000× *g* for 60 min; BCA, TEM, DLS, and immunoblotting for CD9/ALIX/flotillin/Hsp70	100 µL at 1 mg/mL, equivalent to 100 µg EV protein; single administration	Multipoint LV intramyocardial injection after the Dox model was established; exact interval after final Dox dose NR; echocardiography at 2 weeks after treatment and histology at 4 weeks	↑ LVEF/LVFS; ↓ remodeling/fibrosis, ANP/β-MHC/collagen I, apoptosis, inflammation
[379]	Dox-induced cardiac injury/HF, Mouse	Human trophoblast-derived sEVs	300× *g* for 10 min, 2000× *g* for 20 min, 10,000× *g* for 30 min, and 100,000× *g* for 60 min; TEM, NTA, and immunoblotting for CD9/CD63/TSG101	50 µg in 25 µL PBS; single administration	Intramyocardial injection at three sites during the 4 week Dox protocol; exact timing relative to first/final Dox dose NR; echocardiography: 1 week before and 1, 2, 3, and 4 weeks after EV injection; terminal at 4 weeks	↑ EF/FS; ↓ fibrosis/remodeling, inflammatory markers, cardiomyocyte apoptosis
[380]	RIHD, Mouse	Human umbilical cord MSC-derived sEVs	300× *g* for 10 min, 2000 × *g* for 20 min, 10,000× *g* for 30 min, and two 100,000× *g* 70 min ultracentrifugation steps; NTA, TEM, and immunoblotting for CD9/CD63/CD90/TSG101	1 × 10^10^ particles in 100 µL per i.v. injection; five injections, cumulative 5 × 10^10^ particles	Tail vein injection after recovery from local cardiac 20-Gy irradiation followed by 4 alternate day injections; serum: days 14 and 28; echocardiography and terminal histology on day 42	↓ injury enzymes, fibrosis/inflammation; ↑ LVEF/LVFS; improved ultrastructure
[381]	Ang II-induced cardiac hypertrophy, Mouse	Engineered human peripheral blood-derived sEVs	300× *g* for 10 min, 2000× *g* for 10 min, 10,000× *g* for 30 min, 0.22 µm filtration, and two 100,000× *g* 1 h ultracentrifugations; Amicon washing after engineering; TEM, NTA, zeta potential, and ALIX/TSG101/CD81 immunoblot	In vivo sEV/siRNA dose NR; 100 µg/mL sEV and 730 pmol siRNA per 100 µg/mL describe preparation/in vitro loading, not the amount administered to each mouse	i.v. on days 8, 10, and 12 after Ang II pump implantation; terminal histology, echocardiography, and qRT-PCR on day 14	↓ NOX4, HW/BW, fibrosis, cardiomyocyte CSA, ANP/BNP/β-MHC; ↑ LVEF/LVFS
[382]	Aged diabetic cardiomyopathy, 14-month-old rat; 8-week high fat diet plus STZ, followed by 2 further weeks of high fat feeding	Conventional UCMSC sEVs and cytokine-induced VCAM-1+-UCMSC sEVs; VCAM-1 induction used IL-1β, IL-4, and IFN-γ	Differential ultracentrifugation at 3000× *g*, 10,000× *g*, and 100,000× *g*; BCA protein assay, TEM, particle-size analysis/NTA, and immunoblotting for CD9/CD81/TSG101; calnexin control	200 µg exosomal protein per rat in approximately 200–250 µL per injection; three injections; cumulative dose: 600 µg	Tail vein, 3 injections at 1 week intervals; echocardiography and terminal assessment 1 week after the third injection	VCAM-1+ UCMSC sEVs superior to conventional UCMSC sEVs in improving cardiac function/repair; ↓ fibrosis, senescence, ferroptosis, iron/ROS/cTnT
[383]	CVB3-induced viral myocarditis, Mouse	AD-Exo dual-targeting VP1 sEV vaccine with ABD plus DCpep modifications	3000× *g* clarification, 0.22 µm filtration, and 100,000× *g* ultracentrifugation; peptide modified products further purified by 100 kDa ultrafiltration and ultracentrifugation; TEM, NTA, immunoblotting for CD9/TSG101/VP1 with calnexin control, and flow cytometric verification of peptide loading	4 × 10^11^ particles, equivalent to 100 µg total protein per immunization; three immunizations; cumulative 1.2 × 10^12^ particles/300 µg	Subcutaneous injection at the base of the tail every 2 weeks for three doses; CVB3 challenge 2 weeks after the final dose; immune/pathology assessment 7 days after challenge and survival follow-up at 28 days	Dual-targeted vaccine: ↑ humoral/T-cell immunity and dLN/DC targeting; ↓ viral load, cTnI, inflammation/damage; ↑ survival
[384]	CVB3-induced viral myocarditis, Mouse	Human platelet-derived EVs	Clinical-grade patented serial filtration and lyophilization process; PBS reconstitution; NTA/TRPS/zeta potential, SEC-assisted TEM/dSTORM, tetraspanin multiplex, immunoblotting, and ELISA. Products were heterogeneous and contained non-vesicular co-isolates	0.25 mL at 1 × 10^10^ particles/mL (2.5 × 10^9^ particles) per injection; three injections, cumulative: 7.5 × 10^9^ particles	i.p. injections on days −1, 0, and +1 relative to CVB3 infection on day 0; hearts examined at day 10 post-infection	PEV/pmPEV ↓ myocarditis; PEV stronger and ↓ fibrosis/inflammatory markers; no pericarditis effect
[385]	MI, permanent LAD artery, Mouse	P1- and P8-heart tissue-derived EVs	Collagenase II tissue digestion; 300× *g* for 5 min, 2000× *g* for 10 min, 10,000× *g* for 10 min, and 120,000× *g* for 2 h followed by PBS wash/repeat ultracentrifugation; NTA, TEM, and immunoblotting for CD81/CD63/ALIX	NR	Intramyocardial injection on post-MI day 7; echocardiography: day 27; harvest: day 28	No EF/FS or proliferation benefit; P8-EVs ↑ PAK2-ERK1/2 macrophage M1 polarization/phagocytosis; ↓ neonatal CM proliferation
[386]	MI, permanent LAD artery, Rat	Rat BMSCs pretreated with EVs isolated from rat plasma 24 h after MI; EVs were not administered directly	ExoQuick precipitation after serum clarification and 0.22 µm filtration; EVs resuspended in 400 µL and characterized by TEM. NTA and EV marker immunoblotting were not reported	Administered product: 3 × 10^6^ BMSCs in 60 µL; in vivo EV dose NR. In vitro, EVs were examined at 0.5–2 µg/mL	BMSCs injected intramyocardially into two infarct margin sites 3 weeks after permanent LAD artery MI; cell survival assessed at 24 h and 4 weeks after transplantation; echocardiography and terminal outcomes at 4 weeks	↑ BMSC survival, capillary density, EF/FS/LV dimensions; ↓ BMSC apoptosis and remodeling

## Data Availability

No new data were created or analyzed in this study. Data sharing is not applicable to this article.
